# Targeting ferroptosis for leukemia therapy: exploring novel strategies from its mechanisms and role in leukemia based on nanotechnology

**DOI:** 10.1186/s40001-024-01822-7

**Published:** 2024-04-09

**Authors:** Muhammad Hossein Ashoub, Razieh Razavi, Kamran Heydaryan, Masoud Salavati-Niasari, Mahnaz Amiri

**Affiliations:** 1 Department of Hematology and Medical Laboratory Sciences, Faculty of Allied Medicine, Kerman University of Medical Sciences, Kerman, Iran; 2 Stem Cells and Regenerative Medicine Innovation Center, Kerman University of Medical Sciences, Kerman, Iran; 3 Department of Chemistry, Faculty of Science, University of Jiroft, Jiroft, Iran; 4 Department of Medical Biochemical Analysis, Cihan University-Erbil, Kurdistan Region, Iraq; 5 Institute of Nano Science and Nano Technology, University of Kashan, P.O. Box 87317-51167, Kashan, Iran; 6 Student Research Committee, Faculty of Allied Medicine, Kerman University of Medical Sciences, Kerman, Iran; 7 Neuroscience Research Center, Institute of Neuropharmacology, Kerman University of Medical Science, Kerman, Iran

**Keywords:** Ferroptosis, Hematological malignancies, Nanomedicine, Ferroptosis inducers, Novel strategies

## Abstract

The latest findings in iron metabolism and the newly uncovered process of ferroptosis have paved the way for new potential strategies in anti-leukemia treatments. In the current project, we reviewed and summarized the current role of nanomedicine in the treatment and diagnosis of leukemia through a comparison made between traditional approaches applied in the treatment and diagnosis of leukemia via the existing investigations about the ferroptosis molecular mechanisms involved in various anti-tumor treatments. The application of nanotechnology and other novel technologies may provide a new direction in ferroptosis-driven leukemia therapies. The article explores the potential of targeting ferroptosis, a new form of regulated cell death, as a new therapeutic strategy for leukemia. It discusses the mechanisms of ferroptosis and its role in leukemia and how nanotechnology can enhance the delivery and efficacy of ferroptosis-inducing agents. The article not only highlights the promise of ferroptosis-targeted therapies and nanotechnology in revolutionizing leukemia treatment, but also calls for further research to overcome challenges and fully realize the clinical potential of this innovative approach. Finally, it discusses the challenges and opportunities in clinical applications of ferroptosis.

## Introduction

Regulated cell death (RCD) is crucial for both ontogenetic development and homeostasis, and its imbalance can lead to various pathological conditions, including cancer [1, 2]. Apoptosis, first characterized by Kerr et al. in 1972, is the most well-known form of RCD [3]. As many cancer cells rely on the disruption of apoptosis pathways for survival, these pathways have been extensively studied for therapeutic interventions [4, 5]. However, over the past few decades, research has uncovered additional forms of RCD, distinct from apoptosis in their morphological features and mechanisms [6, 7]. Among them, ferroptosis is a non-apoptotic form of RCD that is dependent on iron and lipid peroxidation. Ferroptosis is a unique type of cell death, differing from apoptosis, necrosis, and autophagy [8]. Apoptosis is a programmed cell death involving energy-dependent biochemical mechanisms that lead to specific cell changes and death, such as blebbing, cell shrinkage, nuclear fragmentation, chromatin condensation, and chromosomal DNA fragmentation [4, 9]. Necrosis, on the other hand, is a traumatic cell death resulting from acute cellular injury and is often triggered by external factors like infection, toxins, or trauma [10, 11]. Autophagy is a process where a cell degrades its own components via the lysosomal machinery, acting as a survival mechanism during nutrient deprivation, but it can also result in cell death [10, 12, 13].

Unlike these forms of cell death, ferroptosis is dependent on iron and reactive oxygen species (ROS). It is characterized by diminished or absent mitochondria cristae, a ruptured outer mitochondrial membrane, and a condensed mitochondrial membrane [14]. These abnormalities stem from the loss of selective permeability of the plasma membrane due to severe membrane lipid peroxidation and oxidative stress [15]. Ferroptosis induction is not reliant on the Caspase family, which is essential for apoptosis but is closely associated with lipid metabolism, amino acid metabolism, iron metabolism, and gene regulation. It's important to note that ferroptosis has been connected to various human diseases, including cardiac ischemic disease, kidney disease, liver damage, and degenerative disease [8, 16]. In the context of cancer, the induction of ferroptosis in tumor cells could be a potential therapeutic strategy [17].

The term "ferroptosis" was first introduced in 2012 by Dixon et al. as an iron-dependent form of non-apoptotic cell death induced by the RAS-selective lethal small molecules erastin and (1S,3R)-RSL3 (RSL3). Mechanistically, erastin inhibits the uptake of cystine through system Xc^−^, leading to a depletion of cellular glutathione (GSH). Later, Yang et al. discovered that glutathione peroxidase 4 (GPX4) is a target of RSL3 and a crucial regulator of ferroptosis in many cancer cell types [8, 14, 16]. In particular, triggering ferroptosis might present a new treatment approach for cancer types that prove resistant to conventional methods and apoptosis [18–21].

Moreover, seemingly, the cancer cells acquire a kind of sensitivity to ferroptosis as a way of neutralizing strategy against a variety of targeted treatments, which entails a chance for ferroptosis-inducing therapy (FIT) in relapse management [22]. However, for many hematological cancers, the current treatment methods may result in low therapeutic efficiency, emphasizing an urgent need to investigate alternative treatment modalities. Immunotherapy is a revolutionary cancer treatment that is especially beneficial when leukemia recurs after treatment or when conventional treatments such as chemotherapy fail. However, as a new treatment, immunotherapy does not work for all leukemia types [23–25]. Both ferroptosis and immunotherapy have shown promise in treating leukemia. Ferroptosis is unique in its capacity to target metabolic pathways other than those addressed by immunotherapy. This could provide an alternate or complementary option to current treatments, particularly when immunotherapy fails [26, 27]. In addition, leukemia cells are more likely to express transferrin and contain more iron than other tumor cells, which makes it easier for ROS to accumulate in leukemia cells and trigger ferroptosis. As a result, encouraging ferroptosis via increasing the iron concentration of leukemia cells appears to be a practical technique for leukemia therapy [28–30].

To develop inducing reagents capable of triggering influential ferroptosis beneficial against leukemia ailments, the researcher must develop a comprehensive understanding of the molecular mechanism and the relevant signaling ferroptosis pathways [31]. Scientific research so far has identified several genes, nanomaterials, and tiny molecules capable of inducing the ferroptotic death of cells. However, due to the scarcity of innate iron, simple molecule reagents or genes might not adequately improve the Fenton reaction's effectiveness [32, 33].

Additionally, due to the weak selectivity accompanied by the tiny molecules and genes, the mechanism to avoid undesirable side effects constitutes another hindrance to their clinical applicability. Hence, it could be said that nanomedicine enlightens the path to developing newly devised ferroptosis inducers applicable to cancer/leukemia treatment [34]. As the literature shows, many researchers have reported an undeniable serious relationship between ferroptosis on the one hand and nanomedicine on the other hand, and this has been regarded as an unprecedented strategy to devise nanomaterial-based reagents for outstandingly effective treatment of various cancers; therapeutics like iron-based nanomaterials and those without iron by increasing the ROS level after the cellular uptake, can achieve cancerous cell death, thus resulting in an effective therapy [34, 35].

Nanomedicine strategies using nanoparticle-based compounds for delivering drugs, diagnosing cancer, and inducing cell death, are prospective methods on the near-future horizon [36, 37]. This review offers a summary of nanomedicine's current role and ferroptosis approaches in diagnosing and treating leukemia.

## Modulators of ferroptosis

### Iron metabolism

Among the vital trace elements found in the body, iron can be mentioned; the irregular distribution within the body may damage routine physiological procedures. Fe^2+^ formed through intestinal absorption or erythrocyte degradation Fe^3+^ can be oxidized by ceruloplasmin. It is capable of binding to TF (transferrin) located on the cell membrane so that TF-Fe^3+^ is formed, creating a complex via membrane TFR1 1 (protein TF receptor) to endocytose the same complex [38]. After that, through STEAP3 (six transmembrane epithelial antigens of the prostate 3), Fe^3+^ is reducible to Fe^2+^, and Fe^2+^ remains within the LIP (iron pool) and ferritin, mediated through DMT1 (divalent metal transporter 1) or ZIP8/14 (Zinc-Iron regulatory protein family 8/14). Also, FPN (ferroportin) oxidizes the remaining Fe^2+^ into Fe^3+^ [39]. The iron homeostasis within cells is controlled by internal iron recycling. Ferroptosis induced by erastin [40] can be inhibited by silencing the gene encoding TFR 1 (TFRC). However, HO-1 (heme oxygenase-1) can accelerate the ferroptosis induced by erastin through iron supplementation [41]. HSPB1 (heat shock protein beta-1) can decrease intracellular concentrations of iron through TRF1 expression inhibition [42]. The total amount of iron in an adult human body is ∼3–5 g, up to 80% of which is found within hemoglobin. Less than 20% of the iron is accumulated in macrophages and hepatocytes. Iron deficiency leads to anemia, and excess iron results in hemochromatosis [43, 44]. The cells contain two forms of iron: Fe (III) and Fe (II). The Fe (II) proteins act as catalysts contributing to reduction–oxidation reactions. However, iron is stored and transported in its stable form of Fe (III). Due to the excess iron atoms donating electrons to O_2_ and H_2_O_2_ to create superoxide anions and hydroxyl radicals, both can damage cells through oxidization of nucleic acids, lipids, and proteins. Additionally, using H_2_O_2_ and Fe(II) mixture, it would be possible to oxidize organic matter such as alcohol ester for the creation of reactive oxygen species through Fenton reactions [45]. Higher reactive oxygen species levels can concentrate in tumor cells, and excess amounts of iron are considered a risk factor for the development of tumorigenesis. Therefore, the excess reactive oxygen species created by iron may promote cancer development.

**Fig. 1:**
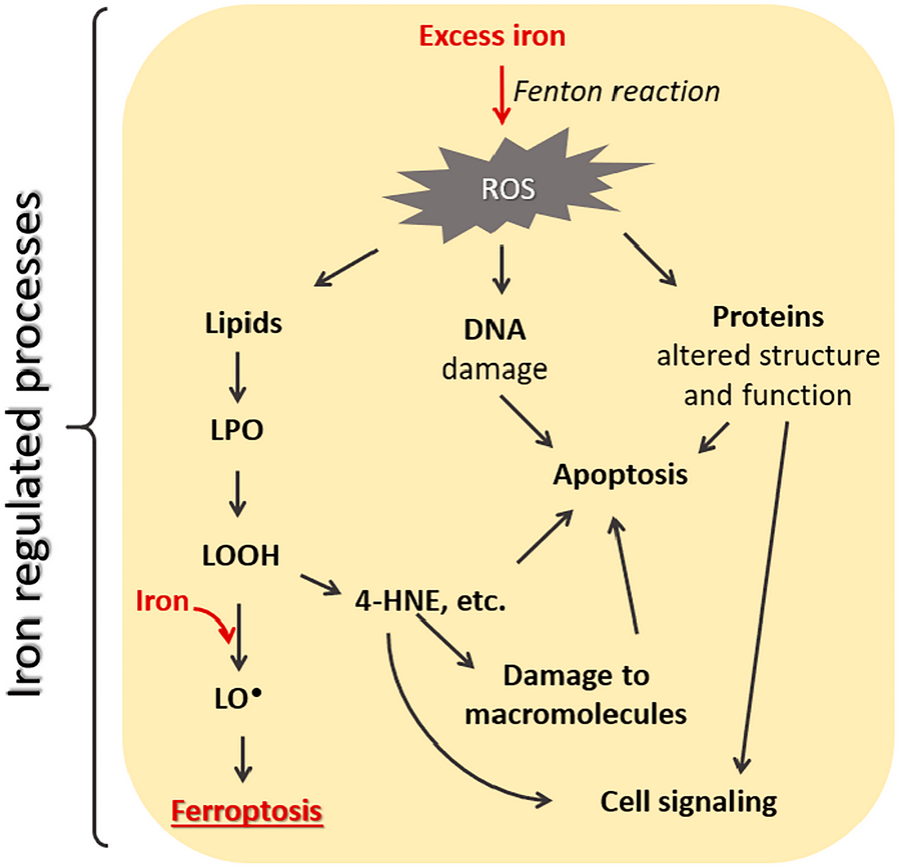
Role of iron in ferroptosis and apoptosis [47]

Nonetheless, the evidence showing that iron-dependent accumulation of reactive oxygen species results in ferroptosis (Fig. 1), the procedure considered responsible for the inhibition of cancer cells, is controversial [46, 47]. Ferroptosis is an iron-dependent procedure that can be prevented via iron chelators. Changes in iron regulation gene transcription, including FBXL5, TFRC, FTL, FTH1, and IREB2, effectively affect the sensitivity of ferroptosis induced by erastin, which shows a high correlation with the iron found within the intracellular area. Also, the higher level of iron found within the extracellular region can lead to sensitization of cells to ferroptosis in vitro and in vivo, and high-iron diets in mice can lead to cellular death [48].

Nevertheless, by keeping glutathione in its decreased form and reducing the iron levels found within the intracellular region, HSPB1 (heat shock protein family B member 1) may prevent ferroptosis [42]. By regulation of the iron abundance level, HO-1 (heme oxygenase 1) and PHKG2 (phosphorylase kinase catalytic subunit gamma 2) can mediate ferroptosis [49]. The three known routes contributing to the iron-dependent accumulation of lipid reactive oxygen species in ferroptosis are (I) reactive oxygen species created by the reaction of Fenton with iron, which is considered a non-enzymatic inorganic chemical reaction; (II) reactive oxygen species created by autoxidation of lipids which is subject to an iron-catalyzed enzymatic control; and (III) reactive oxygen species created due to PUFAs esterification and oxygenation by LOX (lipoxygenase) containing iron atoms. However, while iron's key role in ferroptotic cellular death has been approved, the regulation of ferroptosis by iron remains unknown [50].

### Lipid metabolism

Lipids are key actors in energy storage and the composition of intracellular membrane systems. The PL (phospholipid) oxygenation increases ferroptosis within cells [51, 52]. The accumulation of iron-dependent lipid reactive oxygen species is involved in all ferroptosis pathways. The metabolism of lipids is highly correlation with ferroptosis. Given their sensitivity to lipid peroxidation, PUFAs (polyunsaturated fatty acids) are among the key elements of ferroptosis [53]. It is necessary to oxidize and esterify PUFAs into membrane phospholipids while they constitute the synthetic lipid signal transduction media substrate to transmit the ferroptosis signal. PE (phosphatidylethanolamine) is the major phospholipid-inducing cellular ferroptosis. Phosphatidylethanolamine contains AA (arachidonic acid) or its derivative adrenaline. ACSL4 (long-chain family member 4) and LPCAT3 (acyl-CoA synthetase lysophosphatidylcholine acyltransferase 3) are involved in phosphatidylethanolamine remodeling and biosynthesis and thereby affect the PUFAs transmembrane characteristics and activate PUFAs. Therefore, decreasing the ACSL4 and LPCAT3 expression may decrease the lipid peroxide substrate accumulation within cells and prevent ferroptosis. Eventually, under the LOX (lipoxygenase) catalysis, PUFA-PE plays further oxidative roles and causes ferroptotic cellular deaths [51].

### (Anti-)oxidant metabolism

Lipophilic antioxidants and iron chelators can avoid the accumulation of lipid reactive oxygen species that trigger ferroptosis. NOXs (NADPH oxidases) can provide a source of accumulated reactive oxygen species in the ferroptosis induced by erastin [54]. In Calu-1 cells, erastin-induced ferroptosis was remarkably rescued by inhibiting PPP (pentose phosphate pathway) and NADPH oxidases. Nonetheless, inhibition of PPP or NADPH oxidases in higher erastin concentrations led to partial rescue of the ferroptosis induced by erastin in HT1080 cells. Therefore, the NOX and pentose phosphate pathways seem to be a downstream outcome rather than a starting agent, resulting in inconsistent results for various cell lines. Also, peroxidation products of cell membrane lipids are another source of producing ROS. Polyunsaturated fatty acids are particularly peroxidized in ferroptosis, and after erastin treatment in HT-1080 cell lines, polyunsaturated fatty acids, e.g., arachidonic acid and their derivatives, e.g., linoleate, were significantly reduced [49, 55].

Numerous regulators and pathways in fatty acid synthesis, including citrate synthase, glutaminolysis, and acetyl-CoA carboxylases, are critical for implementing ferroptosis. Arachidonic acid is preferentially acylated by Acyl-CoA Synthetase Long-Chain Family Member 4 (ACSL4), while the insertion of acylated Arachidonic acid into phospholipids of the membrane is preferentially catalyzed by Lysophosphatidylcholine Acyltransferase 3 (LPCAT3), ultimately resulting in lysoPC (Lysophosphatidylcholines) to PC (phosphatidylcholines) conversion. These genes are essential for implementing ferroptosis induced by GPX4 (glutathione peroxidase 4) inhibition. Additionally, Acyl-CoA Synthetase Family Member 2 (ACSF2) is necessary for ferroptosis induced by erastin. These genes guarantee the sufficient production of membrane lipid polyunsaturated fatty acids so that ferroptosis for the next ROS generation and lipid peroxidation is promoted [54, 56, 57]. Through the system Xc^−^ inhibition, catalyzing the deoxygenation of polyunsaturated fatty acids of membrane lipid to produce fatty acid hydroperoxides, lipoxygenases cause ferroptosis. For example, through ferroptosis inhibition, zileuton (a 5-lipoxygenase inhibitor) grants neuroprotection against oxidative glutamate damage. Typically, glutathione peroxidase 4 changes fatty acid hydroperoxides into fatty acid alcohols. However, the above procedure is blocked due to the GPX4 inactivation during ferroptosis. Through Fenton reactions with iron mediation, the accumulated fatty acid hydroperoxides are catalyzed again into harmful lipid peroxyl radicals. Ferroptosis increases the oxidative damage of polyunsaturated fatty acids of membrane lipids. Also, AKR1 (Aldo–Keto Reductase Family 1) expression was significantly increased by the system Xc^−^ inhibition, leading to detoxification of cytotoxic oxidative breakdown products polyunsaturated fatty acids, including MDA (malondialdehyde) and 4-HNE. Nonetheless, the mechanism behind the ferroptosis induced by direct inhibition of GPX4 remains unclear [58].

### Energy metabolism

Given the role mitochondria play in creating ATP through OXPHOS (oxidative phosphorylation), they are vital for most typical cells. Nonetheless, this procedure requires ROS production as an oxidative phosphorylation byproduct [59]. Mitochondria implement various regulated cellular deaths, including apoptosis and autophagy, and play a vital role in the homeostasis of tissues [60, 61]. The experimental ferroptosis induction through xCT inhibition led to the loss of the mitochondrial reactive oxygen species production, MMP (mitochondrial membrane potential), ATP depletion, and induction of mitochondrial fragmentation [62, 63]. The cells were rescued from ferroptosis caused by cysteine or erastin deprivation, supporting the necessity of mitochondrial metabolism in the ferroptosis implementation by mitochondrial depletion or oxidative phosphorylation inhibition via Parkin-mediated mitophagy in vitro [64, 65].

VDACs (mitochondrial voltage-dependent anion channels) are the transmembrane channels transporting ions and metabolites with a critical regulatory role in the ferroptosis phenomenon. Erastin is activated on anion channels with voltage dependency, leading to mitochondria dysfunction and releasing many oxides that eventually lead to cell death caused by iron mediation [66]. According to the triggering ferroptosis strategy, the requirements for mitochondrial metabolism when implementing ferroptosis differ significantly. Whenever launched by cystine starvation or by erastin, leading to glutathione depletion, the mitochondrial TCA activity (tricarboxylic acid cycle) was necessary for the induction of ferroptosis [40]. On the other hand, cancerous cells had deficiency in FH (fumarate hydratase), a metabolic enzyme of the TCA cycle and the suppressor of the mitochondrial tumor, so they could not be subject to ferroptosis through deprivation of cystine [64].

Nonetheless, in the case of pharmacological inhibition of glutathione peroxidase 4, cells were affected with ferroptosis without considering the tricarboxylic acid cycle; therefore, the activity of glutathione peroxidase 4 is essential for ferroptosis inhibition [64]. In line with this idea, mitochondrial damage and ROS production occur during the ferroptosis execution once xCT inhibition or cystine starvation is realized. However, they are unnecessary for the ferroptosis induced by inhibition of glutathione peroxidase 4 [62, 64]. Nonetheless, the AIF (apoptosis-inducing factor), related to mitochondria's inner membrane, translocates from mitochondria to the nucleus, participating in ferroptosis upon GPX4 removal, showing a specific mitochondrial permeability level throughout ferroptotic deaths [67]. Since the cells with deficiency in both proteins of Bcl-2 associated X (BAX) and Bcl-2 homologous antagonist/killer (BAK1) could undergo ferroptosis, this permeability is independent of them [54].

## Mechanisms and pathways of ferroptosis regulation

### GPX4

**Fig. 2:**
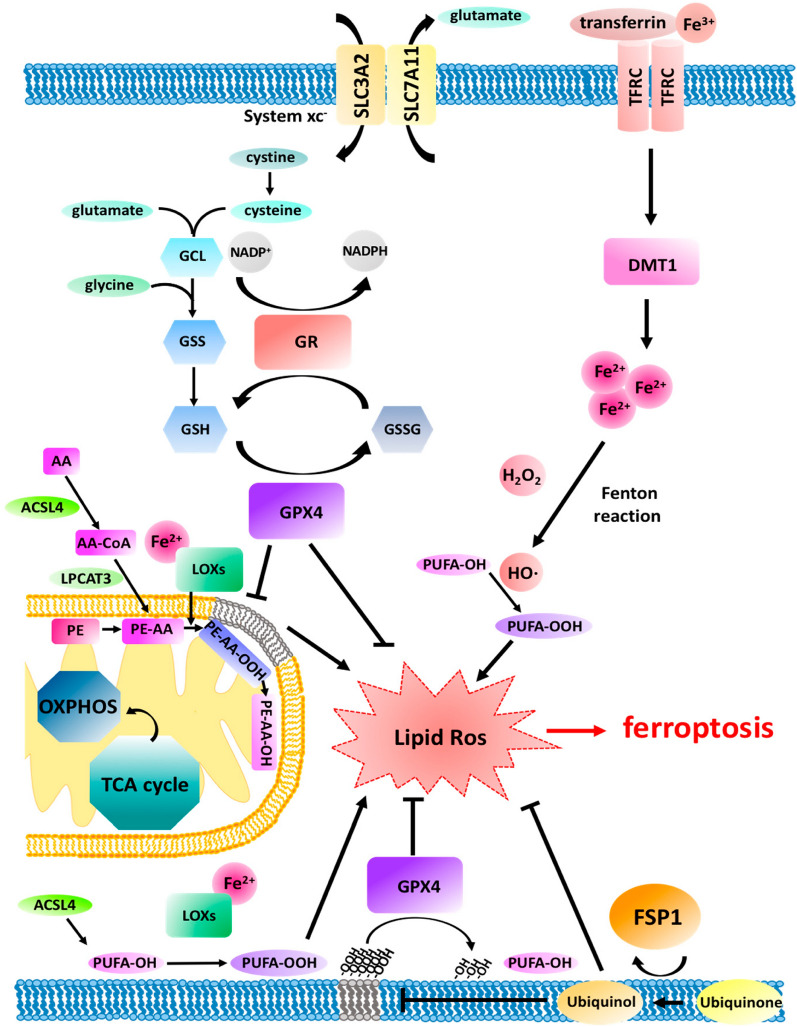
The regulatory mechanisms of ferroptosis in a cell [22]

The GPX4 (antioxidant enzyme glutathione peroxidase 4) is of the glutathione peroxidases family consisting of eight known mammalian isoenzymes, i.e., GPX1-8. By reducing GSH (glutathione), GPX4 catalyzes the reduction of hydrogen peroxide and lipid peroxides. Thus, it acts in line with protecting the cells against oxidative stresses [68, 69]. Also, GPX4 plays a vital role in ferroptosis and is considered the critical regulator of its occurrence (Fig. 2) by preventing lipid peroxide formation [22]. The GPX4 activity prevention results in lipid peroxides accumulation due to the GPX4 role, which reduces the L-OOH (cytotoxic lipid peroxides) to the concerned L-OH (alcohols) and converts glutathione (GSH) into GSSG (oxidized glutathione). Upregulated GPX4 expression prevents ferroptosis, while the down-regulation of GPX4 expression leads to a higher sensitivity of cells toward ferroptosis. RSL3, a ferroptosis inducer, prevents the GPX4 activity directly; thus, it reduces the cellular antioxidant capacity and ROS accumulation, leading to ferroptosis. The compounds DPI10 and DPI7 act on glutathione peroxidase four directly, leading to ferroptosis induction. Selenocysteine is among the vital amino acids of the active group of GPX4. Selenocysteine tRNA is necessary to insert selenocysteine into glutathione peroxidase 4. The MVA (mevalonate) pathway can affect the GPX4 synthesis to regulate ferroptosis by regulating the selenocysteine tRNA maturation. By eliminating intracellular lipid ROS, GPX4 can inhibit cellular ferroptosis. Therefore, GPX4 inhibition launches ferroptosis [68, 70–72].

### Mevalonate

GPX4 is a selenoprotein containing selenocysteine located at the active site, the biosynthesis of which earns isopentenyl pyrophosphate as a donor to modify selenocysteine tRNAs. The MVA (mevalonate) pathway is the critical regulator of the synthesis of isopentenyl pyrophosphate. Therefore, the MVA pathway inhibition could be performed to downregulate the GPX4 biosynthesis, resulting in ferroptosis. Also, the MVA pathway is involved in the production of coenzyme CoQ10 (Q10), also called ubiquinone, which, as an antioxidant in membranes, can resist the lethal lipid peroxidation accumulation and protect cells against ferroptosis. For example, through inhibition of the rate-limiting enzymes of the MVA pathway, 3-hydroxy-3-methylglutaryl-CoA reduced, which was believed to take effect through CoQ10 depletion to reduce GPX4 levels or prevent the biosynthesis of selenoprotein, statin drugs are capable of sensitizing cells to ferroptosis [54, 73, 74].

### HSF1-HSPB1

HSPB1 (Heat shock protein beta-1) is among the small heat shock proteins family and has been lately considered a negative ferroptosis regulator by regulating iron-mediated lipid reactive oxygen species production. By stabilizing the cortical actin cytoskeleton, HSPB1 can downregulate iron uptake by TFR1 mediation, while overexpression of HSPB1 can decelerate transferrin endocytosis and diminish and recycle the intracellular labile pool of iron. The anticancer function of erastin-induced ferroptosis was enhanced in vivo and in vitro by preventing phosphorylation and expression of HSF1 (heat shock factor 1)-dependent HSPB1, which is in agreement with the knockdown effect of HSF1and HSPB1. The HSPB1 overexpression and pretreatment by heat shock prevented ferroptosis induced by erastin [42, 75].

### Nrf2–SLC7A11–HO-1

Nrf (nuclear factor-erythroid 2-related factor 2) is a transcription agent correlated with the cellular defensive response against oxidative stresses in mammalian cells. SLC7A11 and HO-1 (heme oxygenase-1) genes are regulated through the Nrf2 conjugation to the same consensus binding sequence that plays a crucial role in defensive responses against oxidative stresses resulting from the accumulation of reactive oxygen species. The Nrf2–SLC7A11–HO-1 pathway regulates the ferroptosis induced through BAY 11–7085, and heme oxygenase-1 acts as an essential mediator by responding to cellular redox status via the accumulation of iron and regulation of cellular redox. In a linear relationship titled the pathway of p62-keap1-NRF2, NRF2 regulates ferroptosis negatively. NRF2 and p62 bind to Keap1 in a competitive manner. NRF2 interacts with two Keap1 molecules, and the same interaction contributes to NRF2 ubiquitylation and degradation. By increasing the target gene expression in the ROS and iron metabolism, like NQO1 (quinone oxidoreductase 1) and HO1, ferroptosis is inhibited by NRF2. Additionally, the low total survival rate of patients affected with glioma is attributable to higher NRF2 expression, and the NRF2-Keap1 pathway activation can enhance the system Xc^−^ [50, 76, 77].

## Promoting ferroptosis as a novel leukemia treatment strategy

Leukemia, a group of diverse hematopoietic stem cell (HSC) malignancies, is marked by an abnormal buildup of undifferentiated blasts in the bone marrow [78]. These blasts are capable of uncontrolled proliferation, disrupting the production of normal blood cells. Leukemic cells have a unique ability to migrate and invade, with malignant leukocytes retaining the benign leukocytes' capacity for cell motility and survival in circulation while also gaining the ability for rapid and uncontrolled cell division [79, 80]. Recent discoveries in iron metabolism and the newly discovered process of ferroptosis have opened up new possibilities in the field of anticancer therapies [17]. Therefore, targeting ferroptosis could provide new insights into leukemia treatment strategies [81, 82].

### Iron and leukemia

Patients with leukemia often experience a systemic overload of iron, which can be attributed to various factors. The primary cause is the frequent transfusions of red blood cells they undergo, resulting in a substantial accumulation of iron throughout the progression of their illness [83, 84]. Iron overload can also result from ineffective erythropoiesis and a rapid turnover of immature red blood cells, particularly in cases of acute myeloid leukemia (AML) linked with myelodysplasia. From a mechanistic perspective, the suppression of hepcidin due to ineffective erythropoiesis leads to an increased uptake of iron [85, 86]. The process of hematopoietic stem cell transplantation can further disrupt the balance of iron in the body. This happens as it suppresses the production of red blood cells (erythropoiesis) and causes the breakdown of erythroid cells (erythroid cell lysis). These disturbances can have a significant impact on the survival of the patients [87–90]. Compared to normal blood-forming cells, leukemic cells exhibit changes in iron absorption, storage, and release, as well as alterations in the regulatory axis of the iron transporter-hepcidin [91]. Iron and the reactive oxygen species (ROS) it catalyzes are vital for maintaining the balance of the blood-forming system. However, an excess accumulation of iron and a subsequent abnormal surge in ROS can disrupt various biological functions of blood-forming stem cells (HSCs) [92], including their ability to remain dormant, self-renew, and differentiate into multiple lineages.

### Dysregulation of ROS in leukemia

Excessive ROS can induce cell aging and death, impair the ability for self-renewal, and hinder tumor formation. Similarly, an overabundance of iron can alter the tumor microenvironment in a way that encourages cancer cell ferroptosis [93, 94]. Iron plays a critical role in leukemia development because the iron-dependent enzyme ribonucleotide reductase is necessary for DNA synthesis, which supports the rapid proliferation of leukemia cells [95–98]. Moreover, iron overload can trigger the death of neighboring NK cells, CD4^+^ T cells, and CD8^+^ T cells while simultaneously increasing the proportion of regulatory T cells. This allows leukemia cells to evade attacks from the immune system [99, 100]. Excessive iron and ROS can drive the malignant transformation of HSCs by depleting NADPH oxidase (NOX) and glutathione (GSH) [94]. In myelodysplastic syndromes, DNA damage induced by ROS may heighten the risk of leukemia in patients [93]. The research indicates that an overabundance of iron can trigger ferroptosis through the ROS pathway. Moreover, alterations in the iron levels within cells, which are controlled by the ferritin metabolism pathway, have a strong connection to ferroptosis. By managing the equilibrium of iron metabolism, the vulnerability of AML cells to ferroptosis can be modified. Additionally, the advancement of leukemia is associated with a rise in iron accumulation in patients. A recent study by Lopes et al. highlighted the redistribution of iron in acute myeloid leukemia (AML) cells [101]. They discovered that AML patients exhibit increased transferrin saturation (TSAT) and elevated levels of hepcidin, regardless of their history of transfusions. This suggests an increase in circulating iron levels. Using a combination of electron microscopy, energy-dispersive X-ray spectroscopy for elemental analysis, and flow cytometry for labile iron pool (LIP) quantification, they observed an increase in intracellular iron levels in AML cells.

### The role of iron transporters and iron-binding proteins

Compared to healthy bone marrow cells, AML cells demonstrated an increased expression of heme oxygenase-1 (HO-1) and ferritin light chain 1 (FTL1), indicating an accumulation of iron within the cells. The expression level of the iron importer transferrin receptor 1 (TFR1) in leukemia cells was found to be similar to that in healthy bone marrow cells but lower than that in erythroblasts. This suggests that leukemia cells may have a relatively lower demand for iron [101, 102]. However, the relationship between dysregulated iron metabolism and patient outcomes in leukemia remains unclear. A lower expression level of the iron exporter ferroportin 1 (FPN1) in AML cells has been associated with increased sensitivity to chemotherapy and improved patient outcomes [103]. In another study, Trujillo-Alonso et al. found that the expression levels of FPN1 in primary AML blasts and leukemia stem cells were lower than those in healthy bone marrow CD34^+^ hematopoietic stem progenitor cells [104]. They showed that a low FPN1 expression leads to an increase in intracellular iron and oxidative stress, which makes cells more sensitive to the iron oxide nanoparticle ferumoxytol. Collectively, these findings suggest that leukemia cells may have impaired iron flux and an accumulation of intracellular iron, which could potentially make them more susceptible to induction of ferroptosis. The transferrin receptor (TFRC), which primarily manages iron uptake, has been found to be upregulated in acute leukemia [105, 106]. However, its expression level varies among different subsets. For instance, TfR1 is generally higher in Acute Myeloid Leukemia (AML) than in Acute Lymphoblastic Leukemia (ALL) [107], and it's notably higher in T cell ALL than in B cell ALL [108, 109]. Over-expression of TfR1 has been associated with negative impacts on the differentiation of primary blasts [102, 106].

A similar pattern has been observed in lymphoma, where TFRC-regulated iron uptake has been linked to the progression of the disease [110, 111]. This has been used to predict advanced stages in non-Hodgkin lymphoma (NHL) [112]. Furthermore, patients infected with the human immunodeficiency virus (HIV) often develop more aggressive forms of NHL, which is accompanied by higher expression levels of TfR1 [113]. On the other hand, various antibodies that target TFRC have shown anticancer effects against Multiple Myeloma (MM) and lymphoma cells, indirectly explaining the unusual expression of TFRC [114, 115]. Notably, the antigen-binding component of a specific antibody used for detecting ferroptotic cells in vivo is the TfR1 protein [116]. This suggests that TFRC may play a significant role in ferroptosis. Indeed, the cytotoxicity of ferroptosis induced by erastin was significantly reduced by either immunodepleted transferrin or RNA interference of TFRC [40]. Additionally, the recycling of the transferrin receptor (TFRC) and its mediated iron uptake were found to be suppressed by the heat shock response, acting as a negative feedback mechanism in ferroptosis [42]. Regulators related to iron metabolism also influenced the course of ferroptosis by altering cellular iron levels. The Iron Regulatory Protein 2 (IRP2) enhanced its integration to bind target mRNAs, leading to an increase in the intracellular iron pool and sensitizing cells to ferroptosis [54]. Experiments have shown that IRP2 was activated to regulate iron homeostasis and increase sensitivity to ferroptosis through a previously unknown mechanism [117].

Furthermore, Lipocalin 2 (LCN2), which controls iron assimilation independent of transferrin, was associated with iron overload and participated in ferroptosis. The expression of LCN2 in leukemia was complex, with both upregulated and down-regulated conditions observed [118–120]. Interestingly, LCN2 was regulated by a stress-inducible transcription factor to reduce iron accumulation and the resulting oxidative ROS damage, acting as a mediator of resistance to ferroptosis [121]. Ferritin, primarily responsible for storing excess intracellular iron, is composed of two subunits: the ferritin heavy chain (FTH) and the ferritin light chain (FTL). Studies have shown that both FTH and FTL are overexpressed in acute myeloid leukemia (AML), irrespective of genetic anomalies. This dysregulation accelerates pathological development [122, 123]. High serum ferritin levels or FTL expression are both considered potential prognostic markers in lymphoma and multiple myeloma (MM) [124–128].

Interestingly, basal ferritin levels have an inverse correlation with iron toxicity, which enhances the effect of bortezomib on MM cells. Therefore, high ferritin levels could indicate resistance to bortezomib [129]. Ferritin has a strong association with ferroptosis. The process of ferritinophagy, which is activated at the onset of ferroptosis, is mediated by NCOA4. Inhibiting NCOA4 reduces ferroptosis, while overexpressing NCOA4 promotes it [130, 131]. Furthermore, erastin-enhanced ferritinophagy, through FTH overexpression and time-dependent labile iron pool (LIP) alterations, can be blocked by bafilomycin A1 (BafA1), a recognized autophagy inhibitor [49].

Ferroportin (FPN), the only known iron efflux pump in vertebrate cells, has been found to be underexpressed in leukemia and MM [103, 132]. Hepcidin, which regulates the process of iron efflux, plays a role in the tumor evolution of leukemia and MM [133, 134]. Notably, the down-regulation of FPN allows for an increase in intracellular iron, promoting oxidative stress and cell death [104].

In summary, hematological cancer cells tend to have a relatively higher level of iron uptake and storage but a lower level of iron efflux. This results in an elevated intracellular Fenton reaction and increased sensitivity to ferroptosis [135].

### Acute lymphoblastic leukemia

Pediatric hematology has made significant progress in treating acute lymphoblastic leukemia (ALL) through dose-intensification chemotherapy and Allo-SCT. However, the high toxicity risk in chemotherapy, particularly in adults, has led to skepticism about the proper treatment method. Research shows the advantages of pediatric-inspired strategies, but it is uncertain if most adult patients can tolerate the programmed dose intensification. Older patients are more susceptible to dose-limiting toxicities and are often excluded from Allo-SCT. New therapies targeting off-target elements are promising, but a single agent is unlikely to treat all patients [136, 137]. Nevertheless, given the potential of characterizing the individual patient’s leukemia immune genotype and phenotype, the targeted therapy method offers the expectation of improvements in preserving and survival as a stage of the patient-specific treatment approach [138].

After investigating the possible role of ferroptosis in Ph-negative B-ALL using clinical data and RNA-seq results from 80 Ph-negative B-ALL patients, a prognostic model was developed. This model is based on 8 Ferroptosis-related genes (FRGs)—ALOX15, ATP5G3, CARS, CDKN1A, LPCAT3, SAT1, SLC1A5, and TFRC [139]. A study by Lukas and colleagues reported that the inhibitor of Glutathione (GSH) Peroxidase 4 (GPX4), RSL3, induces lipid peroxidation, reactive oxygen species (ROS) production, and cell death in ALL cells. Notably, LOX inhibitors, such as the selective 12/15-LOX inhibitor baicalein and the pan-LOX inhibitor nordihydroguaiaretic acid (NDGA), have been found to protect ALL cells from RSL3-induced ferroptosis [140]. Artesunate (ART), a compound commonly used for malaria treatment, has been found to have strong effects against Adult T cell Leukemia/Lymphoma (ATLL). It does this by inducing the production of reactive oxygen species, leading to cell death through apoptosis, ferroptosis, and necroptosis [141]. A study by Greco and colleagues reported that sulforaphane induced ferroptosis in U-937 cells by depleting glutathione (GSH), reducing GSH peroxidase 4 protein expression, and causing lipid peroxidation [141].

PAQR3, also known as RKTG, is known to act as a tumor suppressor in various human cancers. Jin and Tong demonstrated that PAQR3 inhibits proliferation and enhances ferroptosis in ALL by modulating Nrf2 stability, suggesting that PAQR3 could be an effective biomarker for ALL treatment [142]. Hydnocarpin D (HD), a bioactive flavonolignan compound, shows promising anti-tumor activity. However, the accumulation of lipid ROS and the decrease of GSH and GPX4, along with the inhibition of autophagy, hinder ferroptotic cell death [143]. Poricoic acid A (PAA), a major chemical constituent found on the surface layer of the Poria Cocos mushroom, has protective effects against various diseases. PAA treatments have been found to induce ferroptosis in T-ALL cells by reducing glutathione (GSH) levels and increasing malonaldehyde (MDA) content, thereby inducing autophagic cell death and ferroptosis [144].

Yang and colleagues provided the first direct evidence that circ_0000745 promotes glycolytic metabolism and cell cycle progression while suppressing ferroptosis and apoptosis of ALL cells via the miR-494-3p/NET1 axis. This suggests that the Circ_0000745/miR-494-3p/NET1 axis could be a novel potential target for the treatment and diagnosis of ALL [145]. Another study found that FBXW7 was capable of degrading VDAC3 by modulating cell ubiquitination to promote erastin-induced ferroptosis during ALL. This could explain the potential regulatory link between ferroptosis and autophagy. Furthermore, Zhu and colleagues demonstrated the effectiveness and impact of combining erastin and Rapa to manage ALL both in vivo and in vitro. Zhu and colleagues conducted tests on erastin-induced ferroptosis in ALL cell lines, finding that most T-ALL cells had a poor response to erastin treatment in vitro. They discovered that the upregulation of the voltage-dependent anion channel 3 (VDAC3), mediated by autophagy, promotes ferroptosis. Moreover, the activation of autophagy by rapamycin was found to enhance the anti-leukemia effects of erastin in vivo [146]. Recently, a comprehensive whole-genome CRISPR knockout screen of 7 B-ALL cell lines revealed the system Xc^−^–GSH–GPX4 axis as a common therapeutic vulnerability in B-ALL. This is partially due to the low levels of GSH and FSP1 in these cells [147]. Targeting the system Xc^−^–GSH–GPX4 axis with RSL3, erastin, or sulfasalazine effectively induced ferroptosis in B-ALL cells in vitro. Another study reported the down-regulation of FSP1 and GSH dependency in ALL cells, which was associated with hypermethylation of the FSP1 promoter [148].

Additionally, the redox signal controls the simulation of cell death by the second mitochondrial activator of caspases (SMAC). Additionally, RSL3, an inhibitor of GPX4, or erastin, an inhibitor of the cystine/glutamate antiporter, might team up with SMAC to imitate BV6 and cause ALL cells to undergo ROS-dependent cellular ferroptosis [149]. ALL is highly susceptible to ferroptosis, which is characterized by an excessive buildup of ROS and an elevated amount of lipid peroxidation and is compatible with the already recognized classical mechanism of ferroptosis [140, 143, 149–151]. Overall, these investigations provide a fresh understanding of ferroptosis’ regulatory mechanisms and may help create new therapeutic approaches to revive programmed cell death in ALL.

### Acute myeloblastic leukemia

The long-term survival rate in acute myeloblastic leukemia (AML) patients remains poor, with elderly patients having a higher risk of adverse cytogenetic profiles. Treatment-related higher death risks often prevent optimal treatment, and new therapies targeting off-target elements are introducing promising solutions. However, due to AML's molecular diversity, targeted treatments like FLT3 tyrosine kinase inhibitors may not provide a magical solution [152]. Devising new therapeutic methods accompanied by enhanced genetic profiling and risk classification is a promising approach to gain higher achievements in lowering fatality and increasing survival [153]. A new era of development of newly devised agents is yet to come, where multifunctional nanoparticles can help better respond to patients suffering from relapsed or refractory illnesses and weak cytogenetic characteristics [154]. It has been discovered that AML is susceptible to substances that induce ferroptosis [28, 155–159].

Interestingly, an increasing number of studies have shown that ferroptosis, a form of regulated cell death, is closely related to the pathophysiology of AML. This has shed light on the study of AML pathogenesis and the search for new therapeutic targets. The first study of ferroptosis in acute myeloid leukemia (AML) was conducted by Yu et al. in 2015. They demonstrated that the system Xc^−^ inhibitor erastin induces ferroptosis in HL-60 cells in vitro [160]. The cell death observed was a combination of ferroptosis and necroptosis, as it was prevented not only by ferrostatin-1 and deferoxamine, but also by necrostatin-1 and the knockdown of receptor-interacting protein 3 (RIP3). They also showed the involvement of autophagy and p38 signaling, the inhibition of which reduced the anti-leukemia effects of erastin.

Later, it was shown that erastin-induced ferroptosis in HL-60 cells depended on the cytoplasmic translocation of high-mobility group box 1 (HMGB1) from the nucleus. Knocking down HMGB1 attenuated ferroptosis in vivo [161]. Recently, Pardieu et al. reported that the xCT gene SLC7A11 is a potential therapeutic vulnerability, especially in NPM1-mutated AML, and a poor prognostic factor [162]. The researchers showed that sulfasalazine, an inhibitor of the system Xc^−^, reduced glutathione (GSH) levels and triggered cell death due to oxidative stress. This cell death was partially through a process known as ferroptosis, as evidenced by its partial prevention by a compound called ferrostatin-1. They also discovered that combining sulfasalazine with two chemotherapy drugs, daunorubicin and cytarabine, had a synergistic effect in fighting leukemia. This was demonstrated in a model using cells from a patient (a patient-derived xenograft model) and in primary AML cells. Currently, a clinical trial (NCT05580861) is being set up to test the combination of sulfasalazine and intensive chemotherapy in patients with AML. Also, Cunningham et al. showed that while the use of sulfasalazine to inhibit the cystine-glutamine antiporter proved ineffective when used alone, its efficacy in inducing AML ferroptosis was enhanced when combined with L-buthionine-sulfoximine (BSO) and the induction of cell death across a variety of AML cell lines and patient-derived primary AML samples was significantly enhanced by combining sulfasalazine with BSO to stimulate ROS production [163].

A range of molecular and pathological changes related to ferroptosis have been observed in experimental AML models and AML patient samples. Among the ferroptosis-related genes (FRGs), GPX-1, GPX-3, GPX-4, and GPX-7 were found to be highly expressed in AML patient samples and were associated with a poorer prognosis for overall survival (OS) [157]. AKR1C2 and SOCS1 have emerged as promising biomarkers for predicting prognosis in AML patients [164]. Other markers of ferroptosis were identified among the 12 FRGs (PHKG2, HSD17B11, STEAP3, HRAS, ARNTL, CXCL2, SLC38A1, PGD, ENPP2, ACSL3, DDIT4, and PSAT1) and used to generate a prognostic model. This model stratified patients into low-risk or high-risk groups [165]. Another study unified 18 signature genes (DLL3, EFNB3, ZSCAN4, ASTN1, FAM155B, CCL23, ZFPM2, FOXL1, HMX2, LGALS1, LHX6, PCDHB12, MXRA5, HRASLS, TMEM56, PRINS, TWIST1, and ZNF560) to develop a prognostic risk-scoring model. With the help of this model, AML patients could be grouped into high-risk and low-risk groups, with low-risk patients consistently showing better survival than high-risk patients [166]. Therefore, the development of effective activators and inhibitors targeting ferroptosis could provide new treatment strategies for AML patients.

Pollen Typhae extract contains typhaneoside (TYP), a flavonoid with potential biological and pharmacological effects, such as increasing intracellular and mitochondrial ROS levels when used to treat AML cells. At the same time, TYP caused iron-dependent ferroptosis in AML cells, which was followed by mitochondrial malfunction. By encouraging the activation of AMP-activated protein kinase (AMPK) signaling, TYP also dramatically induced autophagy in AML cells, which led to the breakdown of FT, the buildup of ROS, and, eventually, cell ferroptosis. When taken as a whole, this work offers convincing proof that TYP may be a useful therapeutic drug to stop the spread of AML by increasing cellular ROS generation and ferroptosis [159]. Due to its potential to activate the phosphorylation of AMPK, it has been shown that dihydroartemisinin (DHA), a derivative of the natural medicine artemisinin, can cause ferroptosis in acute myeloid leukemia (AML) cells. The subsequent autophagy-dependent degradation of FTH protein and the release of significant quantities of free iron cause ferroptosis in AML cells due to AMPK’s inhibition of the mTOR pathway and the promotion of autophagy [167]. Prior investigations discovered p53-mutated proteins in individuals with myelodysplastic syndrome and AML. By encouraging the binding of p53 mutants to DNA target sites and reactivating their transcriptional activity, APR-246, a promising new therapeutic drug, can stop the growth of cancer cells. APR-246 has also been found to cause p53-independent cell death in solid tumors. Also, iron-chelating drugs, lipophilic antioxidants, and lipid peroxidation inhibitors reduced early AML cell death following exposure to APR-246, causing an abnormal buildup of lipid peroxides and confirming ferroptosis. Therefore, cells exposed to APR-246 can sustain GSH production by boosting cysteine absorption and stopping cells from creating lipid peroxides. These findings unequivocally demonstrate that APR-246 promotes early cell death in AML by ferroptosis and that APR-246 may, in vivo and in vitro, synergistically increase cell death with inducers of ferroptosis via pharmacological compounds or gene inactivation of SLC7A11 or GPX4 [156, 157]. RSL3, a small molecule inhibitor that targets GPX4, may also cause other programmed cell deaths, such as ferroptosis in AML cells, and it improves the tumor-suppressive effects of first-line chemotherapy medicines (cytarabine and adriamycin) on AML cells [160]. Similarly, NRF2 may be a potential target for AML therapy. According to Balasubramanian et al., NRF2 inhibitor brusatol may decrease NRF2’s capacity to remove ROS and raise the sensitivity of cytarabine and daunorubicin to AML [168].

These findings highlight the potential of targeting ferroptosis in the development of new treatment strategies for AML.

### Mixed-lineage leukemia-rearranged leukemias

The rearrangement of the mixed-lineage leukemia (MLL) gene, found on chromosome 11q23, is noteworthy and frequently occurs in hematological malignancies [169]. From infancy through maturity, MLL gene rearrangement-positive acute leukemia (AL) can manifest as ALL or AML. These leukemia patients exhibit distinct clinical and biological characteristics, such as a high white blood cell count, resistance to standard chemotherapy, a low incidence of complete responses (CR), a poor rate of survival, and a worse prognosis in patients under the age of one year [170]. The World Health Organization has designated it as 11q23/MLL leukemia, a unique subtype of leukemia [171]. Menin-MLL inhibitors, such as MI-463, have the potential to cause ferroptosis in leukemia cells unintentionally. The MI-463-induced decrease in the number of live cells was nearly entirely reversed by ferrostatin 1, but Z-VAD-effect FMK’s on cell death could have been minimal. In addition, DFO and FT inhibitors might stop the synergistic activation of cell death. Therefore, by generating ferroptosis, menin-MLL inhibitors (such as MI-463) may be a valuable strategy for treating MLL [172].

### Chronic lymphocytic leukemia (CLL)

Chronic lymphocytic leukemia (CLL) is the most common leukemia in adults in Western countries, with over 15,000 newly diagnosed cases and 4,500 deaths annually. CLL involves the proliferation and accumulation of mature CD5-positive B cells in the bone marrow, lymph nodes, and blood. Rapid changes in CLL management are occurring, with targeted and non-chemotherapeutic medicine like obinutuzumab, ibrutinib, idelalisib, and ABT-199 transforming current treatment approaches [173–175]. There have not been many studies that mention ferroptosis in CLL. Since human CLL cells cannot convert methionine to cysteine, extracellular cystine absorption is crucial for developing and proliferating these cells. The expression of SLC7A11 is down-regulated in CLL compared to other systemic solid tumors. A decreased capacity of system Xc^−^ accompanies this to transport cystine, which might increase intracellular ROS and ultimately lead to membrane lipid peroxidation and cell death, which could imply a close connection between ferroptosis and CLL [176]. Recent studies have highlighted the prognostic value of ferroptosis-related genes in chronic leukemia. For example, Gong and colleagues suggested that these genes could be used to categorize CLL patients based on overall survival. They also developed a risk signature comprising eight ferroptosis-related genes to predict the overall survival of CLL patients [177]. Ferroptosis, a form of cell death dependent on autophagy, is influenced by BECN1, which affects the initiation and progression of autophagy. Variations in BECN1 expression and repeated allelic deletion have been observed in tumors [178]. Gong and colleagues proposed a novel FPS model for predicting CLL prognosis and identified nine ferroptosis genes associated with CLL prognosis [177]. Human lymphoma and leukemia cells cannot convert methionine to cystine metabolically, so their growth and proliferation must be mediated by the extracellular uptake of cysteine. Interestingly, SLC7A11 was found to be down-regulated in CLL compared to other systemic solid tumors. This reduction in the systemic xCT capacity for cystine could increase the intracellular ROS level, suggesting a strong association between CLL and ferroptosis [176].

### Chronic myelocytic leukemia (CML)

A myeloproliferative neoplasm stemming from myeloid CD34^+^/ CD38^−^/CD90^+^ progenitors in the bone marrow (BM) causes chronic myelocytic leukemia (CML). As a result of the fusion of the Abelson murine leukemia (ABL) genome, chromosome 9, having the breakpoint cluster region (BCR) gene on chromosome 22, and the subsequent expression of an oncoprotein named BCR-ABL, the pathogenesis of CML occurs [179–181]. Several studies have demonstrated the potential of inducing ferroptosis for the treatment of chronic leukemia, especially for aggressive malignancies resistant to conventional therapies [177, 182–184]. Cysteine metabolism, which plays a crucial role in cancer cell proliferation and survival, has been a focus for decades. Depletion of cysteine has been shown to inhibit cancer growth and induce ferroptosis in tumor cells. In particular, K562 chronic myeloid leukemia cells were tested for ferroptosis induced by cysteine depletion. The study showed that simultaneous inhibition of thioredoxin reductase 1 (TXNRD1) by auranofin leads to ferroptosis in these cells. Interestingly, K562 cells resistant to imatinib (K562/G0 cells) exhibited increased sensitivity to ferroptosis induced by cysteine depletion [185]. Song and colleagues found that ferroptosis was involved in the cardiotoxicity induced by imatinib mesylate (IMA) during the treatment of CML. They confirmed that IMA could downregulate Nrf2 expression but upregulate P53 and TfR expression, thereby increasing cellular ROS and iron levels. This evidence suggests that ferroptosis plays a role in IMA-induced cardiotoxicity and highlights ferroptosis as a new target in patients exposed to IMA [186].

### Lymphoma

#### Diffuse large B cell lymphoma

The most prevalent hematologic cancer is DLBCL. The molecular heterogeneity of DLBCL continues to provide a significant treatment challenge despite the current development of novel targeted medicines. The two primary subtypes of DLBCL, aggressive activated B cell-like (ABC) and germinal center B cell-like (GCB) have distinct gene expression profiles and mutation patterns [187, 188]. According to corresponding data, ferroptosis induction was shown to be the mechanism through which dimethyl fumarate (DMF) exerted a broad anti-tumor impact on both DLBCL subtypes. Because of the interaction between low amounts of GSH and GPX4 and high levels of arachidonate 5-lipoxygenase, DMF produces lipid peroxidation in cells, which causes ferroptosis, notably in GCB DLBCL. Overall, the investigation of DMF provides new alternatives for treating DLBCL [189]. However, in the therapeutic management of DLBCL, the ferroptosis inducer sulfasalazine (SAS) can limit GSH formation by inhibiting SLC7A11 transport, indicating the significance of ferroptosis in DLBCL [190].

Because the generation of GSH is inhibited when cells lack cystine, redox imbalance, and ferroptosis results, cystine is an essential negative regulator in the ferroptosis system. As a result, it has been thought that the inability of lymphocytes to manufacture cystine represents a significant advance in treating lymphoma. Early research by Gout et al. showed that the system Xc^−^ inhibitor sulfasalazine might be utilized to dramatically slow the growth of DLBCL in the abdominal cavity of rats [191]. Based on the standard Xc^−^ inhibitor erastin, Stockwell et al. improved imidazole–ketone–erastin (IKE) to have excellent metabolic stability and water solubility. By introducing nanoparticles, IKE might suppress the development of DLBCL in mice with more effective therapeutic effects [192].

Additionally, according to Stockwell et al., suppression of GPX4 activity increased the mortality of DLBCL cell lines [68]. GPX4 has consistently been shown to suppress ferroptosis and decrease lipid peroxides, and its overexpression has been linked to a bad prognosis in DLBCL patients [71]. Additionally, in SU-DHL-8 and WSU-SLCL-2, two DLBCL cell lines, erastin, and RSL3, increased the production of lipid ROS and caused ferroptosis, whereas the administration of the antioxidant vitamin E slowed down the process [72]. Recent clinical research [71], in addition to the aforementioned in vitro studies, has demonstrated that the expression rate of GPX4 in DLBCL patients was 35.5% (33/93) and that the overall survival and progression-free survival of the GPX4-positive group were worse than those of the GPX4-negative group. The ability of GPX4 to lower intracellular lipid peroxidation levels and lessen cell vulnerability to ferroptosis can be used to explain the phenomenon. Collectively, it implies that modulating GPX4 and system Xc^−^ multiple pathways to promote intracellular ROS buildup might improve the vulnerability of lymphoma cells to ferroptosis. It could offer a new line of inquiry for choosing medications for the therapeutic management of hematologic malignancies.

#### Burkitt’s lymphoma

The B cell cancer Burkitt’s lymphoma (BL) has three subtypes: endemic, sporadic, and immunodeficiency-associated BL [193]. Of these three varieties, endemic BL has distinctive characteristics that are frequently linked to the presence of Epstein–Barr virus (EBV). Because there are currently few effective therapy alternatives for BL patients over 60, there is an urgent need to research cutting-edge treatment plans. An improved growth inhibitor for BL has been discovered, and its chemical name is artemisinin [194–196]. In BL cells, DAUDI and CA-46, as a result of activating the ATF4–CHOP–CHAC1 pathway, degrading intracellular GSH, and inducing a stress response in the endoplasmic reticulum, artesunate reduced the ability of lymphoma cells to resist ferroptosis while inducing ferroptosis in BL cells. The protective effects of LIP-1, FER-1, and DFO on cells provide evidence for this effect [197]. Also, p53 might indirectly stimulate ALOX12 (Arachidonate 12-Lipoxygenase, 12S Type) lipoxygenase activity by suppressing SLC7A11 transcription and the system Xc^−^, which results in ROS-induced ALOX12-dependent ferroptosis. Therefore, by controlling SLC7A11’s transcription level and activity, p53 can control the degree of ferroptosis. Removing one TP53 allele also significantly accelerated the development of classical E-Myc lymphoma in xenograft tumor models of E-Myc lymphoma, whereas the loss of one ALOX12 allele inhibited p53-mediated ferroptosis and removed p53-dependent tumor growth inhibition. This implies that ALOX12 is essential for p53-mediated ferroptosis. Additionally, a malignant mutation in the ALOX12 gene can prevent human tumor cells from oxidizing PUFAs and cause p53-mediated ferroptosis [198].

#### Multiple myeloma

One of the incurable hematologic cancers, multiple myeloma (MM), causes a variety of tissue and organ damage due to aberrant plasma cell proliferation at different locations in bone marrow [199, 200]. Some of its clinical characteristics are elevated serum monoclonal immunoglobulin, osteolytic destruction, and anemia accompanying bone marrow infiltration. MM makes up 1.8% of all US malignancies, especially in the elderly [201]. The important ferroptosis regulators GPX4 and SLC7A11 are significantly expressed in MM cells. By decreasing GPX4 and SLC7A11 mRNA and protein levels in U66 cells, a new immunosuppressant called fingolimod (FTY720) can increase ferroptosis. This enhances ferroptosis and autophagy via the PP2A/AMPK pathway [202]. In addition, high proteasome activity in MM cells determines how well-misfolded IgG is degraded to support predicted survival.

**Fig. 3:**
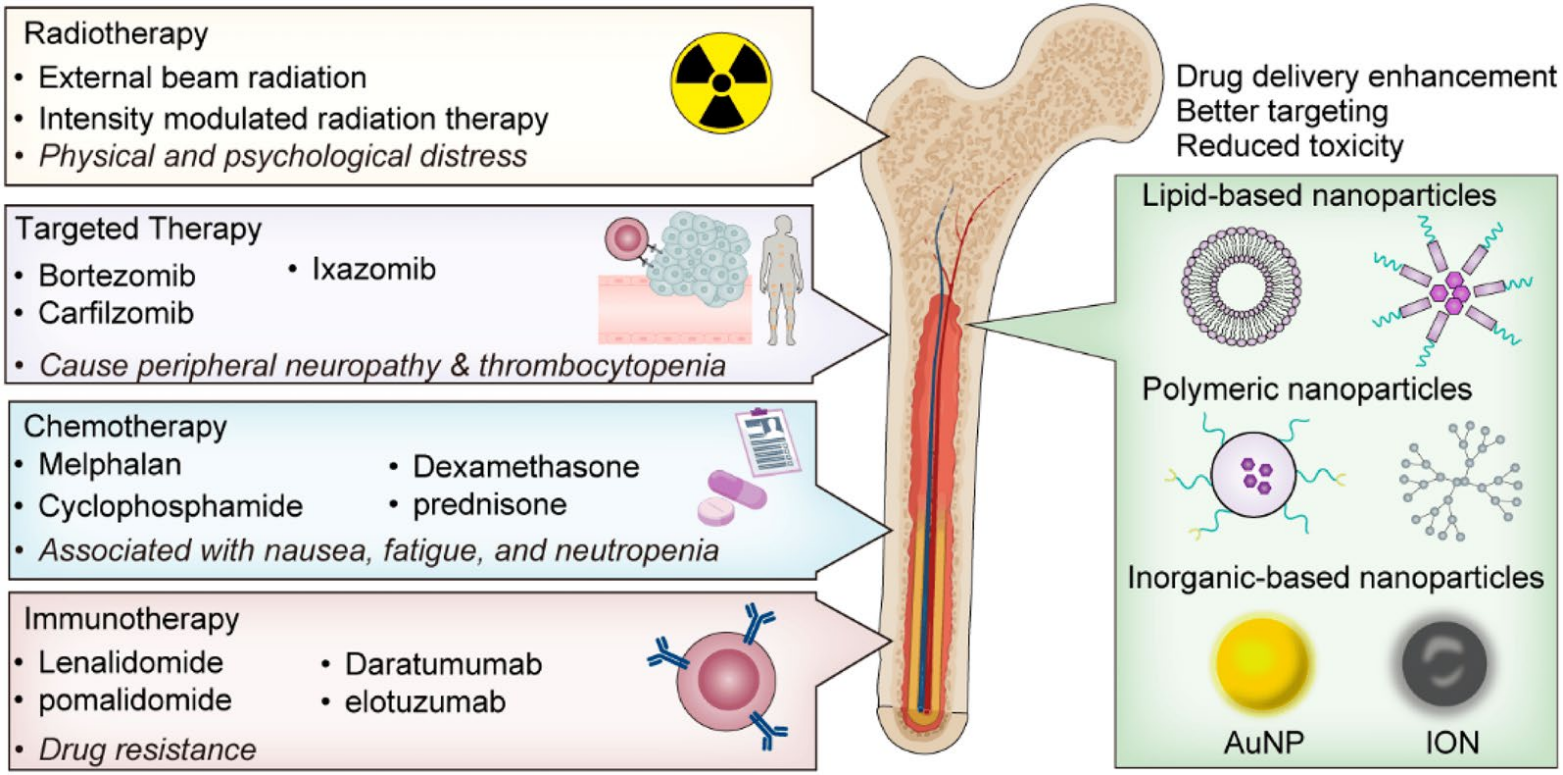
Exploring the present therapeutic strategies for multiple myeloma and the prospective role of nanotechnology [207]

Bortezomib-based chemotherapy has been used in clinical trials to treat MM patients as a proteasome inhibitor [203]. However, the autophagy process, triggered by the buildup of immunoglobulin misfolded in cells, demonstrates the MM’s resistance to bortezomib [204]. Studies have demonstrated that iron exposure can decrease the activity of the proteasome, boosting the effectiveness of bortezomib and carfilzomib (the second generation of proteasome inhibitors used for MM treatment) in MM cells and encouraging ferroptosis, which causes severe MM cell death [205]. Docosahexaenoic acid or eicosapentaenoic acid was used in conjunction with bortezomib to increase the sensitivity of MM cells to bortezomib in order to combat the drug resistance of bortezomib [206]. These combined therapeutic outcomes, in combination with nanotechnology, offer a new theoretical framework and treatment plan for overcoming MM resistance to bortezomib and advancing clinical care (Fig. 3) [207]. Iron is a necessary nutrient that, as was already noted, may hasten the growth of tumor cells. Excess iron is also hazardous since it promotes ROS production [208]. Plasma cells may be highly susceptible to too much iron through the creation of antibodies, the synthesis of a lot of H_2_O_2_ and other byproducts, and finally, activating the Fenton reaction to increase their production of ROS [209]. Thus, causing an excess of iron may reduce the growth of malignant plasma cells and increase the effects of bortezomib, slowing the course of the disease. In 2017, Bordini et al. [209] performed in vitro studies in which they cultivated several MM cell lines (MMCL) in vitro in the presence of high dosages of ferrous ammonium citrate (FeAC) in comparison to untreated and non-MM cell lines, which served as controls. All cell lines showed a lower tendency of proliferation, which was noted. Iron also induced cell death in every MMCL but not in control cells. Raising FT and TF and lowering TFR1 or CD71 may remove extra iron from cells [210]. MYC overexpression [211], which leads to high expression of TFR1 [209, 212], is often found in MM patients. This ensures excess production of iron in the BM microenvironment at least throughout growth by maintaining high iron transporter levels. Iron toxicity must be avoided while the increasing intake is increased to encourage proliferation for MM PC to grow. In other words, the buildup of iron and ROS in MM cells may be essential in illuminating the mechanism of ferroptosis, and the modulation of iron content may eventually change the vulnerability of MM cells to ferroptosis by influencing intracellular ROS homeostasis.

### Inducing ferroptosis using existing drugs

Hypomethylating agents, which are used as a standard treatment for elderly patients with acute myeloid leukemia (AML) and for patients with myelodysplastic syndromes (MDS) [213], have been shown to induce ferroptosis and necroptosis in MDS-derived primary cells and cell lines. One such agent, decitabine, downregulates GSH and GPX4 while inducing ROS and cell death. These effects can be blocked by ferrostatin-1, necrostatin-1, and z-VAD-FMK, indicating that ferroptosis is at least partially involved in the effect of decitabine [214].

In a phase 2 trial, Xiaoyu Liu and his team explored the potential anti-leukemia effects of a combination therapy involving granulocyte-colony stimulating factor (G-CSF), thrombopoietin (TPO), and low-dose chemotherapy in elderly patients with acute myeloid leukemia (AML) [215]. Their findings revealed that TPO triggers ferroptosis by inhibiting EP300-mediated GPX4 transcription, while G-CSF induces pyroptosis via neutrophil elastase, which in turn activates Gasdermin D (GSDMD) in AML cells [216].

In 2020, a study by Birsen and colleagues demonstrated that APR-246, regardless of its presumed effects on mutant TP53, triggers ferroptosis in AML cells during the initial stages of drug exposure [156]. This ferroptosis-inducing effect of APR-246 was later validated in esophageal cancer cell lines, which exhibited an increased turnover of glutathione (GSH) and a suppression of mitochondrial iron–sulfur cluster biosynthesis via NFS1 [217].

Imetelstat, a novel telomerase inhibitor currently being tested in a phase 2 clinical trial for AML (NCT05583552), has been shown to stimulate the synthesis of phospholipids containing polyunsaturated fatty acids (PUFAs) in an ACSL4- and FADS2-dependent manner, leading to ferroptosis in AML cells both in vitro and in vivo [218]. However, it remains unclear whether the induced ferroptosis is a result of telomerase inhibition or off-target effects of the drug [82].

Neratinib, a tyrosine kinase inhibitor approved by the U.S. Food and Drug Administration in 2017 for breast cancer treatment, has been found to trigger autophagy-dependent ferroptosis, G0/G1 arrest, and apoptosis in HL-60 cells. A phase 1/2 clinical trial of neratinib is currently underway in pediatric patients with relapsed or refractory cancer, including leukemia (NCT02932280) [219].

## Ferroptosis and immunotherapy

Recent studies have increasingly clarified the complex and ambiguous association between immunity and ferroptosis [8]. Cancer treatment improves or restores CD8^+^ T lymphocytes' ability to exert effector activity in the tumor microenvironment [220, 221]. Cancer treatment causes CD8^+^ T lymphocytes to become activated, and these cells primarily remove tumors by causing cell death via the perforin–granzyme- and Fas/Fas ligand pathways [222, 223]. In contrast to apoptosis, ferroptosis is a kind of cell death that develops due to the buildup of lipid peroxide in an iron-dependent manner [54, 68]. Despite being mechanistically lighted in vitro [51, 224], ferroptosis may be involved in several pathogenic circumstances, according to recent research [225, 226]. Ferroptosis may have a role in cancer immunotherapy and T cell immunity. However, this is uncertain. According to Wang et al. research [227], ferroptosis-specific lipid peroxidation in tumor cells is enhanced by immunotherapy-activated CD8^+^ T cells, and increased ferroptosis adds to the anticancer effects of immunotherapy. Additional research is required to support our hypothesis that immunological escape is associated with anti-leukemia cell ferroptosis, which results in a bad prognosis for patients. It was discovered that ferroptosis-specific lipid peroxidation in tumor cells is enhanced by immunotherapy-activated CD8^+^ T cells and that elevated ferroptosis, in turn, adds to the anti-tumor effects of immunotherapy. It works by inhibiting the production of SLC3A2 and SLC7A11, two components of the glutamate-cystine antiporter system Xc^−^. This prevents tumor cells from absorbing cystine, which encourages lipid peroxidation and ferroptosis in the cells. In preclinical models, checkpoint blockage and cyst(e)ine depletion by cyst(e)inase work together to improve T cell-mediated anti-tumor immunity and cause tumor cell ferroptosis. IFN expression, the CD8^+^ T cell signature, and the prognosis for cancer patients are all inversely correlated with system Xc^−^ expression. Clinical advantages of nivolumab therapy are associated with decreased SLC3A2 expression and elevated IFN and CD8 levels, according to transcriptome studies conducted before and after the treatment. A unique anti-tumor mechanism is T cell-promoted tumor ferroptosis. Combining checkpoint inhibition with targeting the tumor ferroptosis pathway represents a therapeutic strategy [227].

### Ferroptosis for immunotherapy resistance reversal

A notable advancement in oncology during the past ten years has been immune treatment with immune checkpoint inhibitors, which has demonstrated potential success in treating various cancers. However, despite the immune checkpoint inhibitors' proven therapeutic advantages, drug resistance remains a significant concern. Known tumor cell-intrinsic and tumor cell-extrinsic resistance mechanisms to immunotherapy can be distinguished [228]. The properties of tumor cells that hinder immune cell infiltration or activity inside the tumor microenvironment (TME) are referred to as tumor cell-intrinsic factors. The term "tumor cell-extrinsic factors" describes TME elements other than tumor cells that suppress anti-tumor immune responses. Ferroptosis has recently been demonstrated to have a role in T cell immunity and cancer immunotherapy [227]. Suppression of ferroptosis, according to Jiang et al., was a factor in the resistance to anti-programmed cell death 1 (PD-1)/programmed death-ligand 1 (PD-L1) treatment [229]. Research indicates that ferroptosis can both induce tumor cell death and impact immune suppression by altering various facets of the immune response [230–232]. In the tumor microenvironment (TME) of leukemia, particularly acute myeloid leukemia (AML), ferroptosis exhibits dual effects on immune cells. It has the capacity to directly eliminate cancer cells, thus exerting an anti-tumor effect. However, some studies suggest that cancer cell death via ferroptosis might emit signals that reshape the TME, fostering an immunosuppressive environment [233, 234]. This could potentially lead to resistance to immunotherapy. Highlighting this dual role, ferroptosis has been linked to the death of certain leukocyte subsets, leading to a decrease in immune function, which is one of the numerous ways it can influence TME. Furthermore, the ferroptotic process in immune cells within the TME can be manipulated in various ways to either enhance or suppress the anti-tumor immune response, impacting the overall efficacy of the immune system against the tumor [232, 235]. Ferroptosis has also been associated with features of immunity, inflammation, and lipid metabolism within the TME of diverse AML patients, demonstrating its intricate interactions with leukemic progression and the immune landscape [236].

In summary, ferroptosis is a crucial factor in the intricate balance of immune activity within the leukemia microenvironment, with consequences for both tumor suppression and immune evasion tactics. Therefore, it presents a promising, albeit complex, target for potential therapeutic interventions aimed at adjusting the immune response in leukemia treatment. These results suggest immunotherapy resistance may be overcome by inducing ferroptosis.

#### Tumor-cell-intrinsic processes

The activated mitogen-activated protein kinase (MAPK) signaling pathway, loss of PTEN expression, expression of the WNT/-catenin signaling pathway, loss of the interferon-gamma signaling pathway, and loss of tumor antigen expression are a few tumor cell-intrinsic mechanisms that have been identified as contributing to immunotherapy resistance [228]. These modifications make it unable to produce effective anticancer immune responses. According to recent research, triggering immunogenic cell death to activate the adaptive immune system may transform the immunologically cold state into a checkpoint blockade-sensitive state [237, 238]. It is interesting to note that ferroptosis has been shown to be immunogenic. In preclinical models, Efimova et al. developed a unique strategy for activating the adaptive immune system by inducing ferroptosis-dependent immunogenic cell death. Early ferroptotic cells have been shown to produce molecules (such as adenosine triphosphate and high-mobility group box 1) related to damage and to encourage the phenotypic maturation of dendritic cells originating from bone marrow [239, 240].

Additionally, Luo et al. discovered the eat-me signal 1-steaoryl-2–15-HpETE-sn-glycero-3-phosphatidylethanolamine on the ferroptotic cancer cell surface (SAPE-OOH). Additionally, enrichment of SAPE-OOH targeted macrophages' toll-like receptor 2 to promote phagocytosis [241]. When combined, ferroptosis induction in cancer cells may have a similar impact to a vaccine in promoting anti-tumor immunity and overcoming immunotherapy resistance.

#### Tumor-cell-extrinsic processes

Regulatory T cells (Tregs) and tumor-associated macrophages (TAMs), immune suppressor cells found in the TME, also contribute to immunotherapy resistance [228]. According to Quezada et al., the proportion of effector T cells to Tregs in the TME was associated with the outcome of anti-cytotoxic T-lymphocyte antigen-4 (CTLA-4) immunotherapy [242]. Tregs exert inhibitory control on effector T cells that are invading. In this work, the ratio of effector T cells to regulatory T cells (Tregs) increases when CTLA-4 inhibition is combined with GM-CSF-transduced tumor cell vaccination. The presence of Tregs was shown to be related to resistance to anti-PD-L1 immunotherapy in additional research by Oweida et al. It has been demonstrated that restoring anti-tumor immunity involves focusing on Tregs [243]. Notably, a recent study revealed that GPX4 guards against ferroptosis in Tregs. GPX4-deficient Tregs produce interleukin-1, promoting the T helper 17 response, which improves anticancer immunity [244]. TAMs can polarize into two main phenotypes: anti-tumor M1 (TAM1) and protumor M2, which are another class of cells that appear to influence immunotherapy responses (TAM2). In TME, TAM2 is frequently the most prevalent subgroup of TAMs. In order to increase the effectiveness of immune checkpoint inhibitors against pancreatic cancer, Zhu et al. showed reprogramming TAMs by inhibiting CSF1/CSF1R [245]. According to a recent study, TAM1 demonstrated more resistance to ferroptosis than TAM2 because it causes more significant amounts of inducible nitric oxide (NO) synthase (iNOS)/NO•. Inhibiting TAM2 survival without impacting TAM1 through regulating ferroptosis by iNOS/NO• enhanced anti-tumor immunity in the TME [246].

Furthermore, Jiang et al. demonstrated that in a preclinical mouse model and human patients, increased TYRO3 expression was related to resistance to anti-PD-1/PD-L1 immunotherapy. In addition, the polarization of TAM1 to TAM2 was promoted by TYRO3, which also prevented tumor cell ferroptosis. Aside from causing ferroptosis and TAM reprogramming, TYRO3 inhibition also made cancer cells more susceptible to immunotherapy [229]. Reducing immune suppressor cells by inducing ferroptosis in the TME may be a tumor cell-extrinsic strategy for overcoming immunotherapy resistance.

## Ferroptosis and chemotherapy

One of the main treatments for malignancies is chemotherapy; however, during cancer chemotherapy, many processes have given rise to cancer multi-drug resistance (MDR), which has become the primary cause of chemotherapy failure in cancer patients [247]. Studies on successfully treating cancer MDR have grown in number in recent years. There is a promise for overcoming cancer treatment resistance thanks to ferroptosis in the views of researchers [247, 248]. It is presently understood that suppressing xCT and GPX4 can significantly increase the susceptibility of malignancies to gemcitabine and cisplatin (for example, pancreatic ductal carcinoma, NSCLC, and osteosarcoma) [249, 250]. Several other medications that can encourage ferroptosis have the potential to be used in clinical settings.

### Sorafenib

A multi-kinase inhibitor called sorafenib has received clinical approval to treat advanced malignancies [251]. According to studies, sorafenib's anticancer efficacy relies more on generating ferroptosis by suppressing the activity of the system Xc^−^ than it does on decreasing the activity of the system's kinase whether treating HCC, RCC, lung cancer, or pancreatic cancer [76, 251]. However, sorafenib-mediated cancer treatment has been associated with drug resistance in several cancer cell lines. For example, the target gene of metallothioneins-1G (MT-1G) is a biomarker and a contributing factor to sorafenib resistance, according to research on drug-resistant cancer cells [76, 252]. As a result, MT-1G pathway inhibition during sorafenib therapy can lower the likelihood of chemotherapy resistance and enhance therapeutic benefits [253].

### Artemisinin

Artemisinin has therapeutic utility in the treatment of malaria in addition to being lethal to several malignancies. Artemisinin (particularly artesunate and dihydroartemisinin) can cause ferroptosis in cancer cells by boosting ferritin autophagy and initiating cell death [254–257]. This is accomplished by raising the quantity of intracellular free iron. Artemisinin's anticancer effects can be strengthened by iron supplements like holotransferrin [258]. This is because cancer cells have more heme, which promotes artemisinins' ability to target cancer similarly to how they target malaria [259]. Artemisinin has been shown to help treat head and neck squamous cell carcinoma (HNSCC) [254] and acute myeloid leukemia (AML) [255].

### Cyst(e)inase

An artificially created human enzyme called cyst(e)inase may efficiently break down cysteine and cystine (cyst(e)ine) in blood. Prostate cancer and chronic lymphocytic leukemia cells die in vitro and in vivo due to extracellular cystine depletion [260]. Without manifestly harmful side effects, cyst(e)inase-mediated depletion of the cyst(e)ine may cause ferroptosis in pancreatic cells, indicating adequate safety and tolerability [261]. Using cystinase to control extracellular cystine levels might open up new therapeutic possibilities for ferroptosis-based anticancer therapy, particularly when combined with ROS-inducing medications (e.g., doxorubicin, gemcitabine, paclitaxel, 5-fluorouracil, bortezomib).

### Statins

By blocking HMG-CoA reductase, statins (such as fluvastatin, lovastatin, and simvastatin) are a family of medications used to treat hypotension (HMGCR). Statins can increase ferroptosis by increasing selenoproteins (including GPX4) and CoQ10 production by blocking the mevalonate pathway [74, 262]. According to data from recent studies, fluvastatin and atorvastatin may have anti-proliferative effects in malignancies that overexpress the HMGCR [263–265]. To utilize statins more effectively in upcoming clinical research, it may be helpful to have a more precise knowledge of the ferroptosis pathway controlled by cholesterol.

## Nanomedicine in liquid tumors

**Fig. 4:**
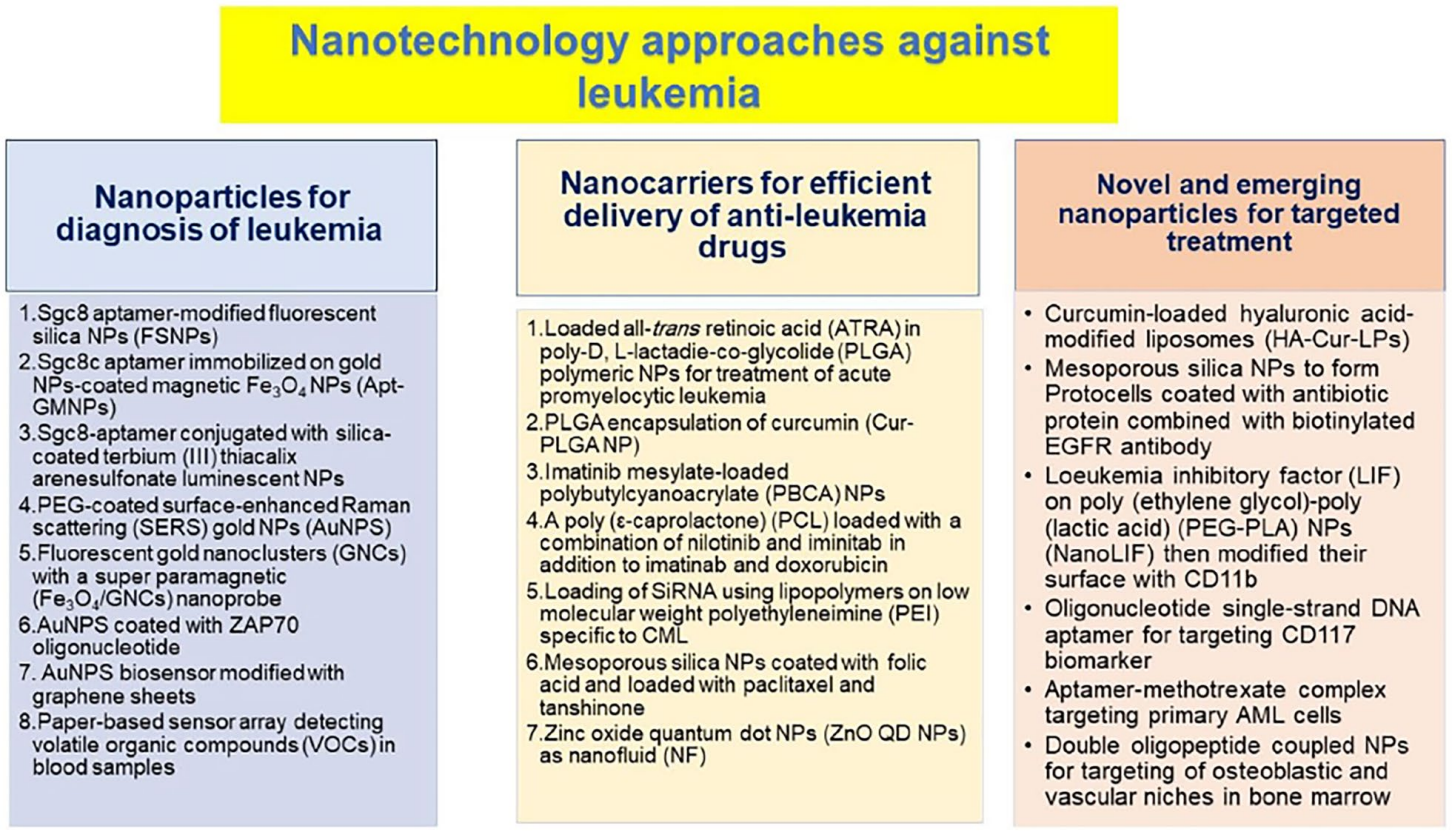
Overview of the utilization of nanotechnology and nanomedicine strategies against leukemia [271]

Nanotechnology-based therapeutics may be advantageous since they are more selective than conventional chemotherapy [266]. These nanosystems not only open up new paths for overcoming the drawbacks of conventional medications, but they also make it possible to combine therapeutic and diagnostic capabilities onto a single platform, advancing nanotheranostics approaches for personalized medicine (Fig. 4) [267–271]. Hematology and oncology are perhaps the two major medical fields within which nanotechnology is most excitingly pursued [272–275]. Conventional chemotherapeutic agents kill both normal cells and malignant in the body and thus cause treatment-related side effects in the patient. On the other hand, the Nanoparticle-mediated targeted delivery of chemotherapeutic agents induces selective apoptosis in malignant cells without harming the normal cells [276].

### Nanomedicine application

**Fig. 5:**
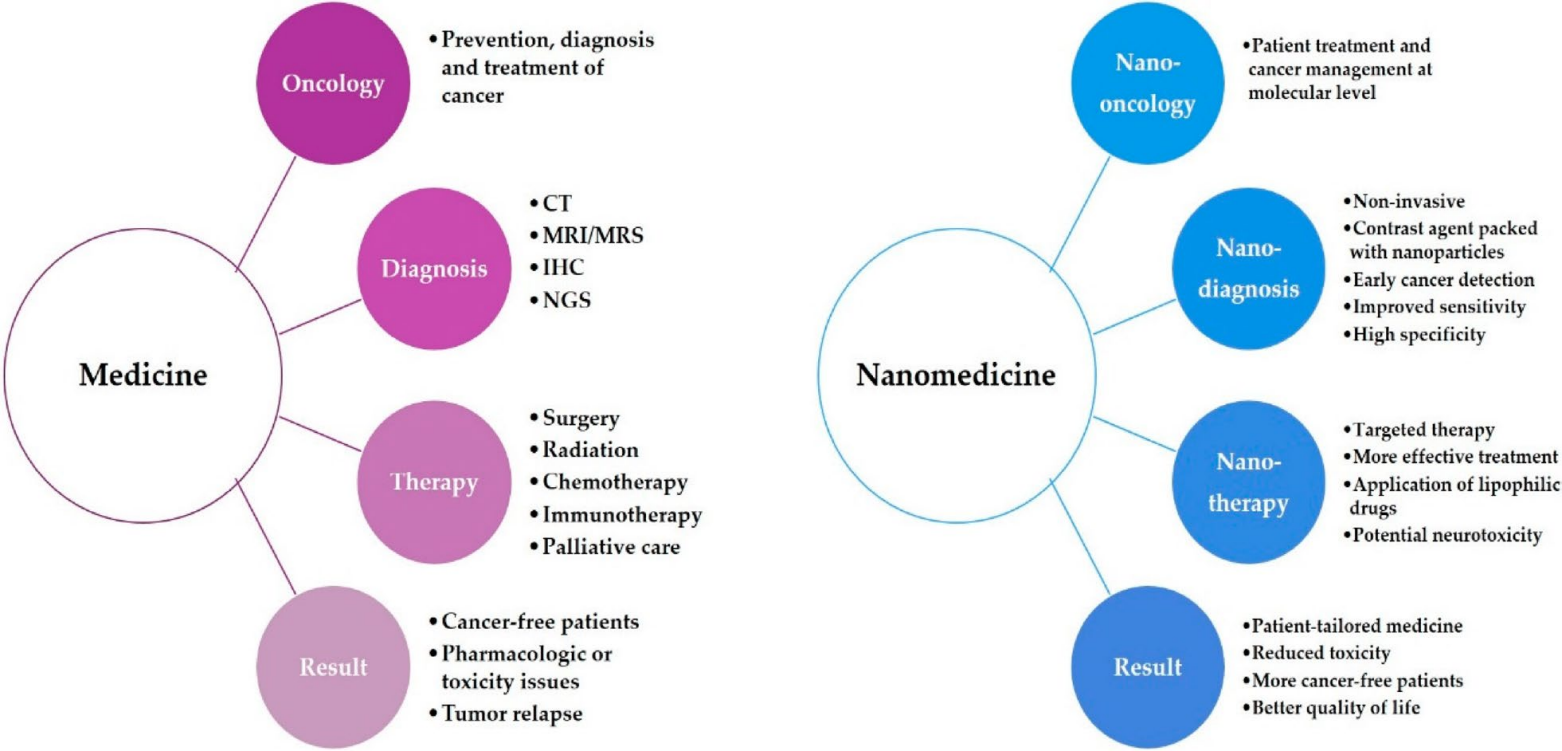
The comparison between traditional medicine and nanomedicine [279]

Nanotechnology is a nanometer-scale configuration of material composition, thereby manipulating their characteristics [277]. The standard sizing of nanomaterial particle design lies between 1 and 100 nm. The application of nanotechnology in the field of medicine is dubbed nanomedicine. Nanomaterials have high surface area-to-volume ratios that facilitate adjusting amounts of targeting ligands to suitable organelle or cell-specific delivery levels and extend the in vivo circulation of hydrophilic polymers via enhancing their “stealth-like” properties. As a result, notable benefits such as (I) heightened solubility of drugs; (II) extended in vivo half-lives of medicinal particles; (III) diminished immunogenicity; (IV) accurate target-bound delivery of drugs; (V) reductions in dosing frequency; and (VI) reductions of dosage, all led to the unparalleled evolution of nano-therapeutics (Fig. 5) [276, 278, 279].

### The function of nanoparticles and nanosystems

Many nanoparticles have already been produced to successfully navigate the bloodstream and anchor to tumor and cancer cells. Additionally, nanomaterials are frequently used in clinical imaging, where their magnetic or optical properties are utilized to screen clinical targets (e.g., tumors at the beginning of their life cycles). In one application, the superparamagnetism of magnetic nanoparticles is utilized in T2-weighted magnetic resonance imaging to facilitate the screening of targeted diseased tissues [280, 281]. In the semiconductor, nanoparticles are utilized via their quantum confinement effects to facilitate multiplexed and ultra-sensitive fluorescence screening in vivo or in vitro. These enhanced imaging procedures grant researchers new and powerful tools to study and comprehend cellular activity and processes concerning various forms of cancer [282, 283]. Another nanosystem is used as a vector for carrying tiny anticancer agents, accurately targeting and delivering to diseased tissue. For example, lipid bilayer-based liposomes with extensive loading capacities are used to deliver clinical agents directly to target sites, resulting in reduced drug toxicity and side effects [284, 285]. An entirely inorganic example is mesoporous silica nanoparticles used as vectors for delivering clinical agents to biological targets [286, 287]. In addition to drug delivery, nanoparticles can be used in a whole host of alternative applications. For instance, nanoparticles capable of transduction convert radio or optical frequencies to thermal energy for therapeutic applications. One example is the coupling of strong near-infrared (NIR) plasmon resonance absorption of gold nanoparticles into thermal energy and subsequent photothermic destruction of malignant tissues [288, 289]. Since multiple systems are required to diagnose and treat cancer, all the nanoparticle-mentioned models are restricted in their efficiency due to their single-purpose design [290].

### Nano-formulations in leukemia treatment

Around 10–20% of AML patients are those with therapy-related AML (t-AML) and myelodysplastic-related AML (AML-MRC) [291, 292]. After being removed from differing clinical trials due to their poor prognosis, a nanotechnology-mediated solution named CPX-351, a liposome-based design consisting of cytarabine and daunorubicin in a fixed 5:1 molar ratio, was ultimately made available for their use in 2017 [293]. Liposome encapsulation of these drugs confers many benefits over conventional approaches, such as reducing toxicity levels, increased in vivo half-lives, and, most importantly, increased upholding of molar ratios that prolong the synergistic effects of administrated drugs and reduce their antagonistic effects [294, 295]. In the second phase of a randomized clinical trial, the efficiency of CPX-351 and conventional 7 + 3 regimens in newly diagnosed AML-MRC and t-AML was evaluated. Despite targeting OS, utilizing CPX-351 raised the median OS when balanced against 7 + 3 regiments (i.e., 9.56 months compared to 5.95 months). Simultaneously, patients treated with CPX-351 showed an increased total remission measure, further establishing the nanomaterial approach's effectiveness [296]. Near one-third of AML patients are correlated to FLT-3 with a poor prognosis. FLT-3, a tyrosine kinase receptor, facilitates propagation, durability, and differentiation blockade in leukemia cells by activating downstream signaling pathways [297, 298]. Polyamidoamine dendrimers affixed to FLT-3 ligands loaded with miR-150 are used as tumor suppressors by directly targeting FLT-3-mutated cells in vivo or in vitro [299]. Another mRNA, miR-29b, is down-regulated in AML and is categorized as another tumor-suppressing agent [300]. Lipopolyplex nanoparticle conjugated transferrin-based delivery of miR-29b to specific targets resulted in the down-regulation of multiple leukemogenesis genes and increased survival rates of mouse models of AML [301]. Nucleolin, a multi-functional protein associated with gene regulation and metabolism, is over-represented on the surfaces of leukemia cells [302]. Gold nanoparticles, such as one loaded with doxorubicin attached by an anti-nucleolin aptamer as an oncogenic micro RNA (AS1411 + anti-miR-221), display exceptional accuracy at targeting leukemic cells with heightened resistance [303]. Acute promyelocytic leukemia (APL) and other AML subtypes are preferably treated with a mixture of all-trans-retinoic acid (ATRA) and arsenic trioxide or alternative chemotherapeutic drugs [304]. A newly developed synthetic retinoid dubbed ST1926 has been designed to target APL and other forms of leukemia. ST1926 displays lowered toxicity and is found to be a more effective treatment for controlling cell growth and promoting cell death. The agent was assembled onto polystyrene-b-poly ethylene diblock copolymer to counteract the lowered bioavailability and fast elimination of ST1926. The resulting structure exhibits excellent effectiveness in reducing leukemic cell proliferation in vitro, reducing tumor burden, and increasing the chances of survival in mouse AML modes [305, 306]. Unlike in typical hematopoietic stem cells, CD33 is usually expressed in leukemic cells and is considered an alternative therapy target. Attachment of anti-CD33 antibody and lanthanide oxyfluoride NP or liposome is a proven way for targeting leukemia cells expressing CD33 [307, 308].

### Combating drug resistance in leukemia

Although nanoparticles and conventional drugs that show excellent effectiveness at destroying bulks of disease populations have been made, an atypical population known as leukemic stem cells (LSC) are shown to be resistant to most therapies and often relapse sometime after treatment [309, 310]. Ineffective treatment of leukemic cells often has the side-effect of LSCs evolving and re-configuring into a new, more resistant population [311]. Such cases demand novel approaches for the elimination of the LSC population. The leukemic stem cell population in AML is heterogeneous, and CD marker expression (CLL-1, TIM-3, CD44, CD47, CD93, CD96, CD123, etc.) differs between patients. Some markers, such as CD44 and CD47, are not exclusive to disease cells and may also be found on healthy stem or progenitor cells. As such, targeting unambiguous AML LSC through varying approaches like cell surface capturing technology and flow cytometry for nanoparticle-agent-assisted targeted therapy is useful [309, 312]. Nano-micelles carrying daunorubicin payloads were designed and attached with a CLI-1 targeting peptide that exhibited an increased lethality towards LSCs and reduced serial transplantation factors in mice models' self-renewal properties of LSCs [313]. Parthenolide (PTL) inhibits LSCs through the upregulation of the p53 tumor suppressor gene and down-regulation of the NF-kβ transcription factor known to promote cell survival. However, due to the poor solubility and bioavailability of PTL, a system where nanoparticles and PTL are carried as payloads on a micelle vector is a great system for targeted elimination of LSCc [314, 315].

### Improving conventional approaches via nanotherapeutic agents

The application of nanotherapeutic agents in the case of leukemia has typically been finding solutions for the problems of low bioavailability and high toxicity in conventional approaches. However, nanomedicine can also be used in several ways to facilitate AML treatment directly: I) Applying nano-based and conventional solutions in tandem for simultaneous destruction of LCSs and bulk disease populations; II) Utilizing nano-based carriers loaded with multiple drugs for combination therapy purposes; III) selectively targeting unique, resistant populations in each individual patient [316]. Treating CML is typically done through utilizing various generations of tyrosine kinase inhibitors (TKIs). However, CML LSCs and domain mutations can sometimes render these treatments ineffective [317, 318]. PEGylated liposome-encapsulated Homoharringtonine (a typical treatment for TKI-resistant patients) has been shown to exhibit less toxicity than the naked drug. In another example, liposome-attached transferrin carrying bortezomib payloads resulted in heightened doxorubicin sensitivity in resistant CML cells [319]. In another case, siRNA loaded on a liposome was applied against BCR-ABL1 and exhibited a decrease in diseases burned in a CML mouse model [320]. Vincristine, a chemotherapeutic agent, is used in several steps of treating ALL and for salvage therapy, despite its high toxicity posing a major issue (e.g., neuropathy that increases in a dose-dependent fashion) [321, 322]. To combat this issue, vincristine sulfate liposome injection (VSLI), a liposome-based design, was developed to increase the half-life and improve the in vivo distribution of vincristine sulfate. The approach allowed for the application of a higher dosage compared to the typical drug. In the second phase of a clinical trial, patients experiencing the second relapse were dosed with 2.25 mg/m2 of VSLI, achieved 20% complete remission, and exhibited a 35% overall response rate [323, 324]. Spleen tyrosine kinase (SYK) is a signal transducer that triggers downstream pathway NF-kβ, and PI3K acts as a central factor of B cell receptor (BCR) signaling in B-ALL [325]. SYK inhibition results in apoptosis and prevents cell growth in leukemic cells. C61 is an SKY phosphorylation inhibitor, as its liposome encapsulation produces a potent anti-leukemic agent [326]. A treatment regimen where the previously discussed approach and total body irradiation (TBI) in low doses can significantly diminish refractory B-ALL clones and result in higher OS when compared to C61-liposome and TBI on their own [327]. CD19 is a trans-membrane protein expressed by B cell lineage, and an increased internalization property due to the conjugation of antibodies makes it suitable for targeting. Additionally, CD19 in the BCR-included complex is a significant element in the proliferation of leukemic cells [328, 329]. Antibody conjugation against C61-liposome marked CD19 exhibited an increased effect on B-ALL eradication in vivo [330]. Attaching anti-CD19 to liposomes carrying a norcantharidin payload exhibits high toxicity and specificity toward CD19-positive cells [331]. Venetoclax is a suitable candidate for treating various leukemia due to its BCL2 inhibitory properties; the increased BCL2 level in patients correlates with therapy resistance [332]. Recombining rituximab and venetoclax in the case of refractory or relapsed CLL cases exhibited excellent efficiency. Additionally, 2-year progression-free survival rates were 84.9% [333]. Applying G3139 antisense to the six initial codons of BCL2 mRNA diminishes chemosensitivity and promotes apoptosis in various cancer cells, such as CLL [334]. Targeting the delivery of G3139 via immune liposome conjugate rituximab has recently proven its therapeutic benefits [335]. Immuno liposomes carrying miR-29b payloads were attached by a tyrosine-proteinkinase transmembrane receptor (ROR1, primarily expressed by CLL leukemic cells) antibody prove to be an effective approach for selective targeting [336].

### Organic nanostructures

**Fig. 6:**
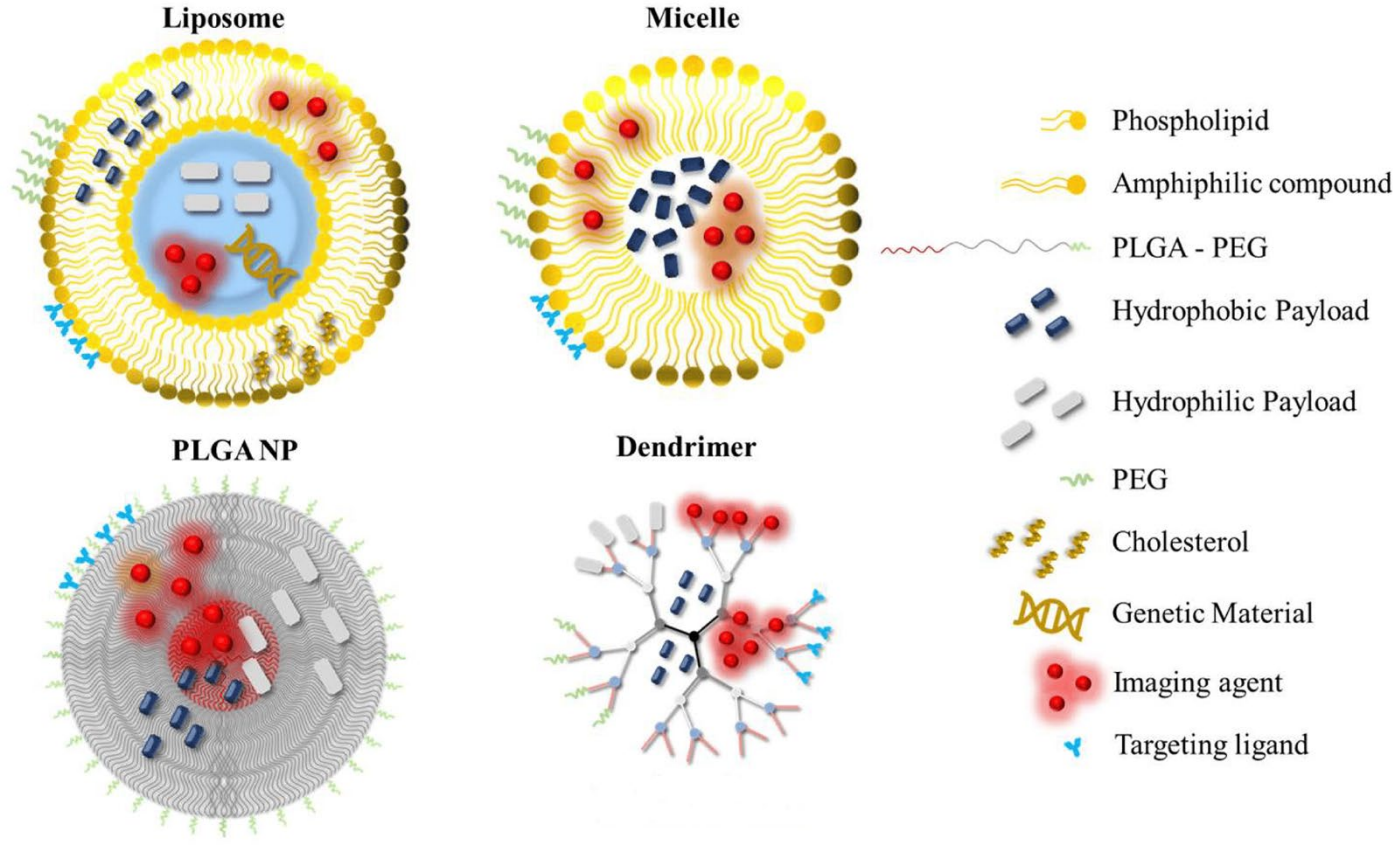
Organic nanoparticles in nanomedicine [337]

The term organic nanoparticle is primarily reserved for polymeric nanoparticles (PNPs) and liposomes (Fig. 6) [337]. The biodegradability and biocompatibility of polymeric nanoparticles, made from synthetic or natural polymers, make them excellent candidates for solutions to many challenges faced in the field of nanomedicine. PNPs can be synthesized through dialysis and two-step emulsification methods, nanoprecipitation, and supercritical fluid technology. These particles' size, solubility, and properties can be modified as needed throughout these processes [338–340]. Liposomes are spherical vesicles that comprise lipid bilayers. They are typically synthesized from cholesterol, surfactants, phospholipids, or proteins made via extrusion and sonication [341–343]. PNPs are primarily known as a delivery mechanism that carries hydrophilic and phobic molecule payloads in their cores and can also be utilized to carry targeted bio-molecules or nanomaterials.

#### Polymeric nanoparticles (PNPs)

Thanks to their capacity for controlled release and extended loading volume, PNPs and liposomes are the most commonly utilized drug delivery elements, especially compared to nanoparticles. Chitosan, gelatin, PLGA, PEG, or block copolymers are the most frequently used polymers in drug delivery strategies [344, 345]. Anti-CD3 antibodies were attached to gelatin nanoparticles and used as a discriminative targeting drug-carrying system for T cell leukemia and T-lymphocytic cells. The outcome showed that only the cells expressing the corresponding TCR receptor exhibited specific internalization of anti-CD3-coupled nanoparticles [346]. An aptamer-membrane protein-based cytosensor, which exhibited sensitive and accurate fluorescence-based detection of acute lymphoblastic leukemia T cells, was recently designed. The detection limit exhibited was as low as 15 leukemic cells [347]. Polymeric micelles of amphiphilic block copolymers are popular nanomaterials used to solubilize imaging agents or hydrophobic drugs [348]. These nanomaterials consist of hydrophobic and hydrophilic polymer blocks that self-assemble in micellar structures, creating a hydrophobic core that retains therapeutic agents [349]. Pluronics are block copolymers capable of enhancing the sensitivity of multi-drug resistance (MDR) tumors to cancer drugs [350]. Other block copolymers such as poly (ε-caprolactone) (PCL) and poly (ethylene glycol) (PEG) are water-soluble block copolymers created as vectors for delivering therapeutic agents such as dexamethasone, a chemotherapeutic often used to treat childhood leukemia. This nanomaterial design exhibited positive outcomes both in vitro and in biological circulation to induce apoptosis of leukemia cells and increase treatment efficacy in mice models [351]. Mouse leukemia models treated with doxorubicin coated in block copolymer nanosystems survived significantly longer, indicating reduced nano-formulated drug toxicity compared to the naked alternative [352].

#### Liposomes

Another family of nanomaterials that exhibit great potential as drug delivery systems is liposomes [353, 354]. They can carry molecules regardless of their affinity toward water [355]. One of the first-ever liposome applications for the delivery of anti-leukemic drugs was cytosine arabinoside encapsulation. The effort enhanced in vivo activity compared to the free drug, significantly improving ALL mouse models' survival [356]. Marqibo®, a vincristine sulfate liposome system, was FDA-approved in 2012 against relapsed Ph-ALL in adults [357], and in the 1990s, preliminary studies were done concerning the pharmacokinetic behavior of this system [358, 359]. Co-encapsulation of cytarabine and daunorubicin) CPX-351( is a liposome-based compound that has been proven effective as an anti-leukemic agent when applied in vivo in five childhood-ALL xenograft models [360, 361].

### Inorganic nanostructures

The term inorganic nanoparticles primarily refers to quantum dots (QDs), metallic nanoparticles (MNPs), and metal oxide (MONPs) nanoparticles. These nanoparticles are semiconductors composed of a shell-coated core, typically attached by bio-molecules like peptides and polysaccharides to increase the particle's general stability in biological systems and combat toxicity by preventing the leaking of heavy metals. Inorganic nanoparticles are expected to have unique physiochemical characteristics and biological consequences that their regular organic counterparts often lack. While inorganic nanoparticles with programmable and varied characteristics offer enormous potential in nanomedicine, their limited clinical application to date is due to non-negligible toxicity concerns in healthy tissues/organs [362, 363]. This age-old problem can now be fully resolved thanks to the development of biodegradable or clearable inorganic nanoparticles [364].

#### Inorganic nanoparticles in the biomedical field

To progress clinical trials and broaden their biological uses in disease diagnostics, a thorough knowledge of the design of these inorganic nanoparticles and their metabolic performance in the body is essential [365, 366]. Inorganic nanoparticles with multifunctional capabilities have been the subject of investigations in recent years to cure various ailments. For use in drug delivery systems, they may be functionalized by various ligands and have distinct physicochemical features [367]. Various inorganic nanoparticles with specific characteristics may be created using various methods. By increasing the bioavailability and release of pharmaceuticals at the targeted region, inorganic nanoparticles offer a wide range of applications in drug targeting [368]. Biocompatible inorganic nanoparticles offer novel platforms to develop cutting-edge diagnostic and therapeutic agents for improved detection and more effective treatment of severe diseases because of their inherent physical properties that may be useful for imaging and therapy, as well as their highly engineerable surface. Although the in vivo use of inorganic nanoparticles has been proven for more than 20 years, it is exceedingly challenging since nanomaterials have extremely complex pharmacokinetic features [367]. As prospective diagnostic and therapeutic systems in the field of cancer, inorganic nanoparticles have recently drawn more interest [369, 370]. Inorganic nanoparticles typically have a variety of qualities that make them ideal for drug delivery to cells, including wide availability, rich functionality, good biocompatibility, the potential for targeted delivery (e.g., selectively killing cancer cells while sparing healthy tissues), and controlled drug release. While organic NPs frequently have high biocompatibility, inorganic NPs have advantages in function and properties [371, 372]. Inorganic nanoparticles exhibit diversity in size and composition-based physical properties compared to organic variants. This makes them more suitable for molecular detection, cell imaging, and other biomedical purposes [373]. Inorganic nanoparticles are seldom utilized on their own and are usually combined with organic particles, which grant them properties such as bio-compatibility, active sites suited for attachment of biological molecules, and solubility in biological media [374, 375]. The most commonly applied nanoparticles in bio-imaging and bio-sensing are gold quantum dots (GQDs), cadmium–tellurium (CdTe), cadmium–selenium (CdSe), indium–arsenate (InAs), indium–phosphate (InP), which are suitable for cellular apoptosis recognition and cell tagging.

#### Metallic nanoparticles

MNPs include precious and magnetic metals such as Palladium (PdNPs), gold (AuNPs), silver (AgNPs), and copper (CuNPs) and are utilized for a broad range of purposes as theranostics. Magnetic MNPs exhibiting extended stability in hypoxic tumor environments are widely used as bio-sensing elements and in contrasting imaging. Iron oxide (Fe_3_O_4_), zinc oxide (ZnO), ceria (CeO_2_), zirconia (ZrO_2_), mesoporous silica nanoparticles (MSNs), and titania (TiO_2_), bio-compatible MONPs, show catalytic and antioxidant effects as well as increased chemical stability. These properties render these constructs suitable as drug vectors and for medical implant and bio-imaging purposes [376–378]. The transcriptome profiles of two leukemia cells (KG1a and HL60) treated with two types of iron nanoparticles (FeNPs and PBNPs) with various ROS regulation properties were presented by Luo et al. [379]. The findings showed that several genes had their expression considerably altered. PBNPs controlled more genes than FeNPs. The distinctive and typical genes were identified. The gene signatures had been identified. Iron metabolism, antioxidation, lipid metabolism, vesicle traffic, innate immune system, and cytoskeleton were among the genes shared by all treatments. Both cells' PBNP levels considerably influenced the pathway for mineral absorption, but HL60's FeNP levels greatly influenced the pathway for lipid metabolism. This work provided fresh information on the cytotoxicity of iron nanoparticles at the level of gene transcription that regulates ROS in leukemia cells with various stemnesses. This work clarified why leukemia cells with low stemness are vulnerable to FeNPs as ROS inducers, whereas those with high stemness are resistant. This work also raised the possibility that stemness leukemia cells could be resistant to iron nanoparticles as an inducer of ferroptosis. Ashoub and colleagues presented a single-step, environmentally friendly approach to create ZnO nanoparticles using *black cardamom* extract. They then exposed healthy cells (PBMCs) and Acute Promyelocytic Leukemia cell lines (NB4 and HL-60) to varying concentrations of these nanoparticles for 24 and 48 hours. The ZnO nanoparticles inhibited the growth of leukemia cells in a time and dose-dependent manner. They promoted ferroptotic cell death by significantly increasing lipid-ROS, intracellular iron, ACSL4, and p53 levels while simultaneously reducing GSH and GPx activity levels. Interestingly, these nanoparticles did not exhibit any toxicity towards healthy cells. Therefore, it can be concluded that ZnO nanoparticles synthesized through this green method can induce ferroptotic cell death in leukemia cells without affecting normal cells [380].

#### Noble metal nanoparticles

The unique optical characteristics of noble metal (gold and silver in particular) nanoparticles, such as their unique surface plasmon resonances, make them widely used in biomedical fields [381]. Thanks to this characteristic, noble metal nanoparticles are preferred over other types, such as magnetic or inorganic semiconductor dots and polymeric nanoparticles. Early-stage cancer could be detected through its biomarkers using a more sensitive system employing gold nanoparticles as surface plasmon resonance. This improves the chances of recovery due to quicker treatment response [382]. The high surface-to-volume ratio of plasmonic active nanoparticles that exhibit strong resonance in near-infrared (NIR) enables them to deliver a large volume of diverse molecules [383–385]. ALL-specific hybrid plasmonic nano-platforms based on silica and nanospheres obtained from sgc8 are used to treat ALL as vectors for delivering paclitaxel drugs into ALL cells (Ramos and CEM). They enable a more effective treatment via intracellular glutathione-triggered drug release [386]. In one gold nanoparticle-based pH-dependent drug delivery system, gc8c aptamer was utilized to enable the capacity for delivering daunorubicin to human ALL T cells (Molt4). The final product exhibited heightened cytotoxicity towards target cells compared to the naked drug and the aptamer–drug conjugate [387]. Detection of CD10 antigen by a quartz crystal microbalance (CM) system employing a gold nanoparticle-based sandwich immunosuppressor [388]. Gold nanoparticles also exhibit anti-leukemia effects, partly through the induction of ferroptosis. For instance, GNR-CSP12 (gold nanorods loaded with chitosan and a 12-mer peptide) can induce ferroptosis by suppressing global m6A RNA methylation. This effect is enhanced when combined with tyrosine kinase inhibitors or PD-L1 checkpoint inhibitors [389]. In the end, multifunctional gold nanoparticles exhibited high efficiency for invading resisting leukemic cells currently under study for various types of leukemia applications Concurrent with the further definition of the role of such new agents and their inclusion into new therapeutic strategies, the adult ALL program probably will follow the pediatric ALL as a critical success in the future [303, 390].

#### Iron-based nanostructures

**Fig. 7:**
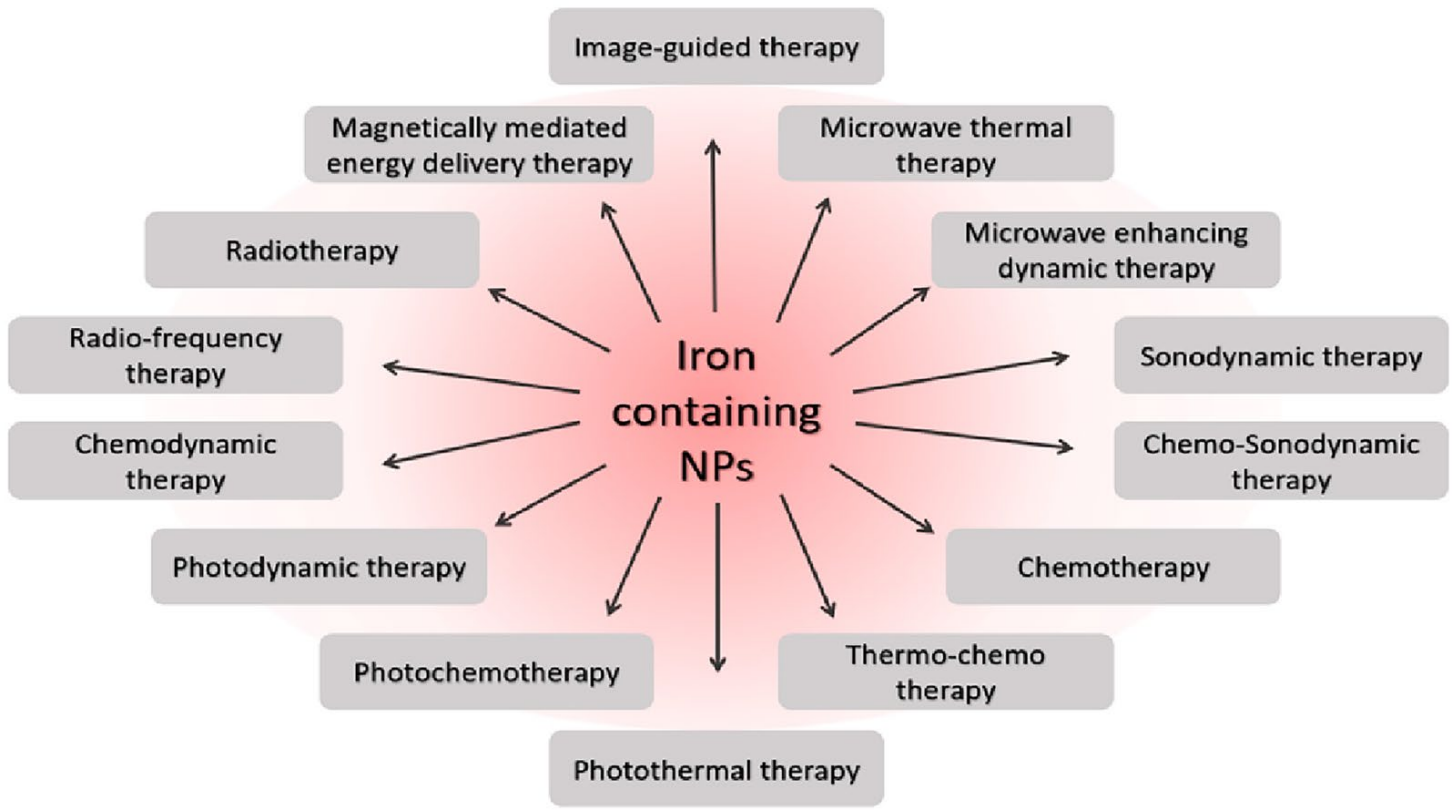
Application of iron-containing nanoparticles in the biomedical field [47]

Iron, the central element in ferroptosis, promotes iron-based nanoparticles as the most logical candidate for use in ferroptotic approaches. Several iron-based nanomaterials can induce ferroptosis and cell death. Examples are iron oxide nanoparticles (IO NPs), iron nanometallic glasses, iron-based up-conversion nanomaterials, iron-based metal–organic frameworks and networks, iron-doped nanomaterials, and iron-based polymer micelles [34]. Iron Oxide nano-particles (IO NPs), FDA-approved nanomaterials, are employed as contrast agents in magnetic resonance imaging, vectors for cancer treatment, and treatment of iron deficiency. Due to their utility, iron-containing nanoparticles are applied in many nanomedicine strategies in oncology (Fig. 7) [47, 391, 392]. It has been recently shown that iron oxide nanoparticles may cause ferroptotic cell deaths. For instance, activatable IO-LAHP nanoparticles were engineered by tethering LAHP (linoleic acid hydroperoxide) on IO NPs. In the tumor sites, the Russell mechanism between Fe^2+^ ions released from iron oxide nanoparticles and linoleic acid hydroperoxide developed tumor-specific singlet oxygen that could inhibit tumor growth by creating ROS, leading to cancer cell death. Therefore, ROS-mediated ferroptosis provides a new cancer treatment strategy [393]. By accelerating the Fenton reaction, the Fe_3_O_4_ NPs can cause ferroptotic cell death by creating ROS. For instance, to demonstrate ROS-mediated cancer therapy via exposure to the US (ultrasound) diagnostic system, some form of H_2_O_2_/Fe_3_O_4_–PLGA polymersome was developed. After exposure to the ultrasound, the H_2_O_2_ encapsulated within the polymersome core will be released and dislocated through the PLGA polymersome disruption to react with Fe_3_O_4_ within the polymersome membrane, thereby generating OH through the Fenton reaction that may cease the growth of the malignant tumors [394]. For fabricating a biodegradable and sequentially functioning GFD (GOD-Fe_3_O_4_@DMSNs) nanocatalyst to induce ferroptosis of higher therapeutic efficacy, GOD (natural glucose oxidase, enzyme) and US Fe_3_O_4_ NPs were integrated into the DMSNs (dendritic mesoporous silica nanoparticles). In malignant tissues, GOD in the GOD-Fe_3_O_4_@DMSNs nanocatalysts can catalyze the glucose into an abundant amount of H_2_O_2_ that, through the Fenton reaction, interacts with Fe_3_O_4_ NPs and generates highly toxic OH that finally leads to ferroptotic cell deaths [395]. Iron has been aimed at being doped in various nanomaterials, given the critical role of iron in ferroptosis for targeted cancer therapy.

##### Iron oxide nanoparticles in the biomedical field

Given that many magnetic nanoparticles, including carbonyl iron, iron oxides, chromium dioxide, nickel, and cobalt ferrite, have been subject to broad studies, iron oxides have been of the most acceptability due to showing the best superparamagnetic characteristics, lower toxicity, the highest surface area for binding drugs, biocompatibility, and narrow size distribution [396–400]. Different coatings can be conjugated, including chitosan, PEI, polystyrene, PEG, dextrans, silica, MNPs, and drugs [401]. In chemotherapy, the modified magnetic nanoparticles have been used in the targeted delivery of agents, including methotrexate, cisplatin, doxorubicin, tamoxifen, and paclitaxel [402, 403]. Iron oxide nanoparticles have been subject to extensive studies in the biomedical field due to their particular magnetic behavior and lower cytotoxicity. Magnetite (Fe_3_O_4_) and maghemite (γ -Fe_2_O_3_) are frequently used for MNP synthesis in vivo and in vitro applications. Other magnetic nanostructures (FeCo, CoPt3, FePt) of metal alloys are only used in vitro due to their significant toxicity to living cells [404–406]. Given their specific physical and chemical properties, MNPs are suitable for different objectives and cell labeling, drug delivery, magnetic resonance imaging, and cell targeting [407–410]. High magnetic saturation, biocompatibility, and appropriate surface functionalization of these nanoparticles are necessary for the above objectives. The nanostructures are aimed at delivering different substances through an active or passive procedure. Given the decreased lymphatic systems of tumor tissues and the permeable vasculature with pore dimensions of 100-780 nm, there is the potential for passive delivery of the targeted drug for tumor tissues [411, 412]. The same characteristics paved the way for the retention effect and improved permeability, allowing higher concentrations of nanoparticles to be delivered at the solid tumor sites. A complex system is required for the active targeted delivery of drugs, indicating the existence of a covalent conjugation of the targeting molecules on the surface of nanoparticles that may be linked to specific ligands expressed in a cancerous cell [413, 414]. Singh et al. (2011) presented their elaborated research on applying paclitaxel-loaded MNPs conjugated with lectin used for leukemia therapy. MNPs are known to be used as drug delivery carriers and avoid in vivo nanoparticle aggregation [415]. Also, MNPs should keep their aqueous dispersibility and magnetic features along with the high drug loading capacity expected profile of drug release and an adequate tolerance of normal cells [416]. By covering the MNPs with glyceryl mono-oleate, one of the polymers featuring a long amphiphilic chain, there could be aqueous dispersibility, paving the way for the incorporation of both kinds of hydrophilic and hydrophobic drugs [417, 418]. The use of specific molecular signatures helps provide suitable therapeutic amounts of drugs at the targeted sites and improves the advantage of endocytosis mediated by a receptor. Additionally, a comparison was made on chronic myelogenous leukemia cell lines between the cellular uptake efficiency for paclitaxel-loaded magnetic nanoparticles conjugated with lectin and for MNPs with entrapped paclitaxel and those with free paclitaxel (K562) [415]. Wang et al. (2011) presented a new formulation of DNRMNPs (daunorubicin-magnetic nanoparticles) for sustained daunorubicin (an anti-tumor agent of the anthracycline class) release. Initially, the oleic acid-containing daunorubicin covered the iron oxide core, followed by stabilizing nanoparticles with Pluronic F-127®. Given the reduced side effects for high daunorubicin amounts of plasma and extended dosing interval of the drug after intravenous injections, the DNR-MNP formulations are highly efficient, making them a good choice for systemic administrations [419].

### Composite nanostructures

**Fig. 8:**
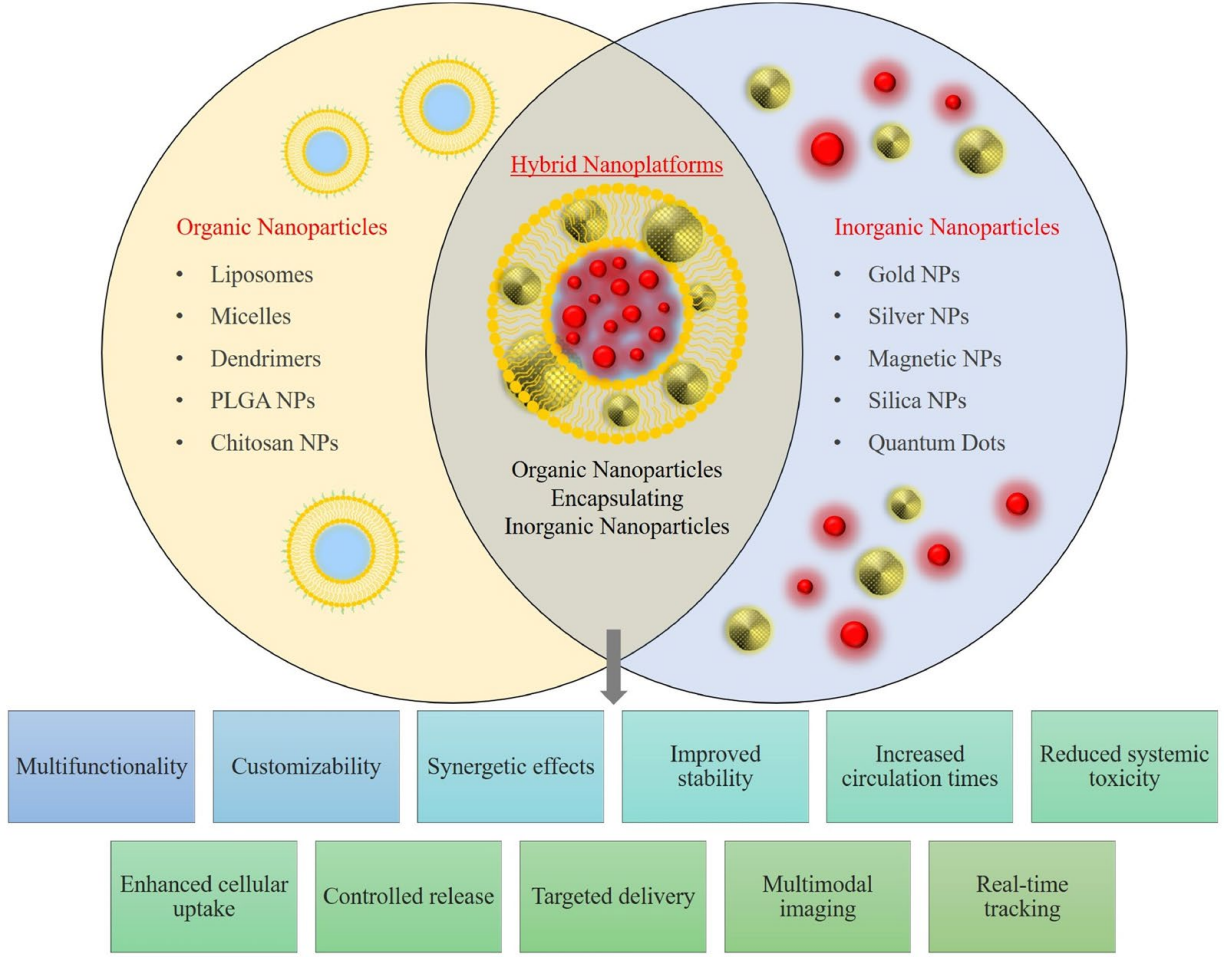
Construction of hybrid NPs via combining organic and inorganic NPs [337]

Polymeric nanostructured materials are polymeric composites or polymer components encompassing nanomaterials [420, 421]. In general, the fundamental polymer matrices in the same systems are interspersed by more than one nanoparticle component, and typically, the hybrid derivatives express unique intrinsic features. Adapting naturally occurring or synthetic polymers such as chitosan, polysaccharides, and collagen as the primary matrix material is often possible [266, 421, 422]. Nanofibers, nanospheres, nanosheets, and nanorods are frequently found within hybrid systems. Hybrid nanoparticles are usually constructed via a combination of inorganic and organic nanoparticles, such as wrapping organic NPs with inorganic NPs or inlaying inorganic NPs in the framework of organic NPs (Fig. 8) [337]. Numerous thermal, chemical, mechanical, and electrical properties could be adapted within the nanocomposite engineering limitations by controlling the inherent material parameters [423, 424]. Although a fundamental perception of an expansive nanostructure range seems necessary for constructing the optimum product, regulated property sequences are attainable in polymeric nanocomposite materials engineering [278, 421, 425].

Polymeric nanocomposites focus on numerous studies as the core bio-applications components, including bio-imaging, implant constituents, drug delivery, and bio-sensing [421, 426]. Although iron oxide (Fe_3_O_4_) nanomaterials can be the most critical particles with superparamagnetic characteristics, given the agglomeration of the magnetic nanoparticles, their usage in biomedical arenas has its disadvantages [427]. To overcome the same challenge, the nanoparticles of Fe_3_O_4_ can be incorporated into the CNTs (carbon nanotubes) [428]. CNTs have been widely used in biomedical fields, given their nano-sized stable tubes, high surface areas, and high aspect ratios [429, 430]. Due to the exceptional properties of MWCNTs (multiwalled carbon nanotubes), such as the enhanced electrical, mechanical, and thermal characteristics, multiwalled carbon nanotubes are among the best alternatives used for controlling the drug molecular release rate. Layla Hosseini (2016) studied the impacts of PLA/MWCNT/Fe3O4 nanofibrous scaffolds loaded with daunorubicin on chronic myelogenous leukemia cell lines (K562) in the presence/absence of external magnetic fields. The findings demonstrated that incorporating daunorubicin into the nanofibrous formulation of PLA/MWCNT/Fe_3_O_4_ under a magnetic field can impose synergistic cytotoxic impacts on leukemic cell lines [431]. Yousefi and Amir-Mohammad (2020) estimated the anticancer property of nanocomposites of ZnO/CNT@Fe_3_O_4_ on cell lines of chronic myelogenous leukemia (K562), which revealed that reducing the proliferative capacity of K562 cell lines would be possible by ZnO/CNT@Fe_3_O_4_ through G1 arrest induction and apoptosis through reactive oxygen species-dependent upregulation of SIRT1and FOXO3a. Also, the pro-apoptotic gene expression was enhanced by nanoformulation of ZnO/CNT@Fe_3_O_4_. However, its inhibitory effect on the anti-apoptotic target gene expression of NF-Κb in K562 cell lines was insignificant. Additionally, a synergistic experiment showed that the same nanoformulation might improve imatinib's cytotoxic impacts on K562 cells. In sum, it seems that the pharmaceutical usage of nanocomposites has promising potential in treatment strategies for leukemia [432].

### Graphene nanoparticles

Another class of nanomaterials studied is the carbon-based one, especially graphene-based designs [433, 434]. Graphene’s special structure grants it a planar aromatic macro-molecule made by a sole layer consisting of a p-conjugated construct of six atom rings, which in turn provide it with great capacity for conjugating large numbers of biological molecules. Since graphene on its own is hydrophobic and thus requires stabilizing agents or surfactants for suspension in biological media, graphene oxide, being amphiphilic, is a better choice for application for biomedical purposes. Graphene oxide also has free functional groups (e.g., hydroxy groups, carboxylic acid, etc.), which enable a good range of functional potential. As such, graphene oxide has been hailed as a great vector for delivering anticancer agents and imaging particles [435, 436]. Graphene was first applied in the field of leukemia treatment for the diagnosis of cancers. The allotrope’s potential in ultra-sensitive (single-cell level) detection of leukemia was documented utilizing electrochemical techniques for three leukemic cell lines, including B cell prolymphocytic leukemia (BCLL) cells and human acute lymphoblastic leukemia (CCRF-CEM), in comparison with chronic myelogenous leukemia (K562) cells. Due to the hyper-activation of nucleotide synthetic pathways in the atypical growth of malignant cells, both graphene oxide nanoplatelets and spongy graphene electrodes were utilized to detect the over-presence of guanine in the leukemic cell cytoplasm [437–439].

### Nanodiagnostics

Morphological examination, immunohistochemistry, antibody microarrays, flow cytometry utilizing fluorescent markers, fluorescence in situ hybridization, PCR, and DNA sequencing are the conventional methods for identifying leukemia and lymphoma cells. Being unable to recognize immature white blood cells at an early stage of the disease is one of the main problems in diagnosing lymphoid and myeloid neoplasms. Effective therapy for these aggressive and frequently occurring cancers rests heavily on the adequacy and sensitivity of the diagnosis [440–443]. A practical method for early detection may include signal amplification in combination with NPs. For example, fluorescent QDs and nanoscale semiconductors have been exploited for improved detection strategies due to their better fluorescent properties. In addition to fluorescence enhancement, researchers have also looked at the distinctive physicochemical characteristics of metal nanoparticles, such as localized surface plasmon resonance (LSPR), photoluminescence, or superparamagnetic properties [444]. An antileukemia-thiolated aptamer (sgc8c) that selectively identifies protein tyrosine kinase 7 (PTK7), an overexpressed transmembrane receptor in human T cell ALL cells, has been proposed for use in aptamer-based nanodiagnostics devices for acute leukemia. Aptamers have been utilized to functionalize AuNPs because of their excellent affinity and selectivity for their targets. With a detection limit of 10 cells/mL and improved detection sensitivity, these aptamer–NP systems can distinguish between leukemic and normal cells [445]. The complex may be employed both in vitro and in vivo, according to Yu et al. [446], who used aptamer-functionalized QDs to identify leukemia cells in the buffer and serum selectively. The toxicity of the complex was also examined in animal models. A specific chromosomal aberration known as the BCR-ABL1 gene, which is exploited for precise molecular diagnostics, is linked to chronic myeloid leukemia. According to a literature survey, gold nanoparticles have been the exclusive focus of CML nanodiagnostics techniques [447]. Colorimetric detection of this molecular aberration has been accomplished on total RNA isolated from blood based on the LSPR of the AuNPs [448]. In order to identify BCR-ABL1 isoforms precisely, Cordeiro et al. [449] used the AuNPs' capacity to alter the fluorescence emission of adjacent fluorophores. The distinction between the e14a2 and e13a2 fusion transcripts enabled the detection of BCR-ABL1 positive samples using AuNPs functionalized with specific ssDNA oligonucleotides. Antigens like CD20, which are overexpressed by malignant B cells, are the main focus of diagnosing leukemia and lymphoma [450].

Rituximab, a CD20 antibody, has been used to treat lymphoma, but it can also be used to diagnose lymphoid neoplasms. In order to selectively identify and separate lymphoma cells from mixed samples, Sahoo et al. [451] employed avidin-modified magnetic NPs (MNPs) functionalized with biotinylated anti-CD20 antibodies and a permanent magnet. For in vivo imaging aimed at the diagnosis of B cell malignancies, Capolla and colleagues also utilized an anti-CD20 antibody functionalized into polymeric-fluorophore Cy5.5-labeled NPs. By loading with medications, these NPs might also be employed as a tailored therapy approach, making them a desirable theranostics platform [452]. The two most prevalent surface proteins produced by B cells and utilized in diagnostic immunophenotyping and CD20 antigens are CD45 and CD19. In an ex vivo model of malignant B cells, MacLaughlin et al. [453] described the use of surface-enhanced Raman scattering (SERS) to detect these three surface proteins. In order to identify target cells with SERS using flow cytometry, AuNPs were functionalized with one of the specified monoclonal antibodies (anti-CD20, anti-CD45, or anti-CD19). This method advances SERS immunophenotyping significantly, increasing the sensitivity and specificity of blood cancer detection.

### Nanotheranostics

Numerous characteristics of the NPs discussed here make them good candidates for nanotheranostics since they are particularly desirable for biomarker identification and simultaneous aberrant cell eradication [454]. Tumor cells are free to move around in liquid malignancies, necessitating deliberate targeting. However, there are also limited tumor locations, such as the bone marrow and lymphoid tissues; in these niches, nanosystems using the EPR effect may be highly relevant. In this manner, therapeutic nanoconjugates may amass in tumor sites, allowing for later active targeting of cancer cells [455]. For instance, 50 nm AgNPs have been attached to p-mercaptobenzoic acid, a Raman sensor and linker molecule, to enable non-invasive detection of live cells without labeling based on SERS rituximab for B cell lymphoma targeting and ablation. Also, the SERS signals from a single molecule were tailored to be amplified by these silver nanostructures, enabling multiplexing. The specially created Ag nanoconstruct could simultaneously identify a single CD20-positive lymphoma cell and eradicate it with remarkable selectivity [456]. Additionally, silica-based diatomite nanoparticles, which have an irregular form and a mean size of around 200 nm, were used to treat B cell lymphoma [457]. These DNPs have been altered to actively target the surface immunoglobulin B cell receptor's very changeable area (BCR) to facilitate fluorescence-based monitoring using FITC and confocal microscopy/flow cytometry. These conjugates also detect and inhibit the anti-apoptotic protein B cell lymphoma/leukemia 2 (BCL2) via siRNA that is included inside them. Target-specific BCL2 gene silencing was accomplished in vitro by the nanostructure. This method might be used to monitor patients with lymphoma/leukemia. Another instance of nanotheranostics has been produced to eradicate B cell lymphoma employing a nano-antibody made of rituximab conjugated to an NP albumin-bound paclitaxel (Abraxane (ABX)) [458]. Alexa fluor 750 labeling of the ABX allowed in vivo imaging of the decreased tumor burden. In vitro and in vivo, the 160-nm noncontract preserved the cytotoxicity of ABX and the CD20 affinity of rituximab. Additionally, compared to ABX or rituximab alone, better therapeutic effectiveness was attained by combining both antibodies at the nanoscale.

### Current limitations

So far, nanoformulations have improved the sensitivity and simplicity of tests to detect biomarkers. The efficacy–toxicity ratio of anticancer drugs has also been proven to be improved by nanomedicines, opening the door to real-time monitoring of liquid tumor diagnosis and therapy. However, the availability of in vivo tumor models that accurately reflect the environment of actual human tumors is crucial for the preclinical success of nanomedicines. Since the pathogenesis of the disease in murine models is not relevant to most human cases, leukemia/lymphoma models face several challenges. Mouse models are essential for studying leukemia and lymphoma, but they struggle to accurately mimic human disease development due to species differences and unique cellular behaviors. The fluid nature of these cancers, complex origin microenvironment, and diverse genetic underpinnings complicate the creation of suitable models. Additionally, these models do not capture the complex microenvironment from which these human cancers arise, as well as their genetic and molecular heterogeneity [31, 459, 460]. These challenges are significant in preclinical research as they may hinder the applicability of the results to human patients. Despite these challenges, mouse models have significantly advanced our understanding of these disorders' pathobiology in humans, aiding in studies on mechanism discovery, oncogenesis, molecular genetics, microenvironment, metastasis, and therapeutic efficacy [31].

Some of these problems are addressed by xenografts. However, they are often performed on immunosuppressed mice to prevent immunological rejection of human cells, ignoring the immune system's impact on tumor growth and the effectiveness and targeting of NPs. Their diminished therapeutic impact is also a result of the variation in experimental circumstances used in the many preclinical trials employing NPs to treat leukemia and lymphoma. For the clinical translation of nanoscale diagnostic tests and treatments, there is a dearth of standardized production practices and controls acknowledged by regulatory agencies such as the US Food and Drug Administration (FDA) or the European Medicines Agency for the clinical translation of nanoscale diagnostic tests and treatments. Determining NPs' capacity as a delivery vehicle, imaging tool, or therapeutic agent requires in vivo toxicity, stability, and biodistribution investigations, which have been lacking [461, 462]. However, the clinical application of nanoscale diagnostic procedures and treatments presents numerous regulatory challenges. If guidelines remain vague, the advancement of nanomedicine could face significant hurdles. Key aspects include identifying key drug product quality attributes, determining appropriate analytical methods, assessing consistency between batches, and ensuring biocompatibility and safety. Additionally, managing intellectual property rights and proving the cost-effectiveness of nanoscale diagnostic procedures and treatments compared to existing therapies are major regulatory challenges. These challenges could restrict the introduction of nanoparticulate nanomedicines (NNMs) into the market despite their therapeutic benefits. Therefore, it is recommended to collaborate closely with regulatory agencies from the early stages of development to ensure alignment and expedite the development of future nanomedicines [463].

## Challenges of clinical translation of ferroptosis

The mechanisms of ferroptosis in overcoming drug resistance in preclinical investigations are outlined in this paper. Before the actual use, there is still a long way to go. First, it is challenging to say if ferroptosis inducers' ability to reverse drug resistance is universal or limited to a small subset of tumors with specific features. Given the wide variation in sensitivity to ferroptosis inducers among cancer cell lines, we must choose a suitable target population that most likely profits from this tactic [73]. This objective will be helped by a greater comprehension of the processes underlying ferroptosis and drug resistance. Second, aside from cancer, degenerative illnesses and ischemia disorders have also been linked to ferroptosis in terms of pathological cell death [464]. Therefore, creating specialized medicines that cause ferroptosis in cancer cells while minimizing side effects on the body will be crucial. Nanoparticle ferroptosis inducers offer particular benefits in this regard [465]. Not to mention, we lack the biomarkers needed to identify ferroptosis in vivo. Investigating appropriate biomarkers will aid in advancing future in vivo studies and clinical monitoring [466, 467]. Ferroptosis still lacks particular indicators, and further research is required to determine its precise processes. To remove barriers and increase the effectiveness of tumor therapy, it is still crucial to understand tumor cell death patterns and drug resistance. Additionally, how ferroptosis works and how illnesses are related must be established [14, 468]. Exist additional ferroptosis regulation routes outside the traditional one, for instance? Is iron required for the catalysis that produces lipid peroxides? Or may other substances take the place of iron in ferroptosis? How may ferroptosis fundamental research findings be used in clinical practice for therapy? These are the issues that require answering. Recent ferroptosis research has generated several novel approaches to treating tumors. As already said, ferroptosis unquestionably contributes significantly to the development and toxicity of hematological malignancies such as leukemia, lymphoma, and MM [469–471]. In order to achieve the goal of killing tumor cells, it is also possible to increase the sensitivity of hematological tumor cells to ferroptosis by controlling the level of ferroptosis-inducible factors, the equilibrium of intracellular ROS production and extinction, and the regulation of iron metabolism homeostasis. Other factors include the intimate relationships that some substances have with ferroptosis in hematological tumor cells and the correlation between ferroptosis-inducible factor levels and the prognosis of hematological cancers.

## Summary and perspectives

Here, we have summarized nanomedicines' current role in leukemia treatment and diagnosis. Through a comparison made between traditional approaches applied in the treatment and diagnosis of leukemia, like Chemotherapeutical approaches and immunological approaches, with some of the most recent nano approaches, we were to emphasize the future is increasing and the potential role played by nanoparticle-based structures (inorganic, organic, composite, and iron-based) in the treatment of leukemia. It has been shown that tumor cells may significantly increase their capacity to fend off oxidative stress by negatively controlling ferroptosis, which may explain how to overcome cancer resistance by activating ferroptosis. Several studies have demonstrated that regulating ferroptosis may overcome resistance to conventional chemotherapy, targeted treatment, and immunotherapy. These results promise the creation of innovative medicines that induce ferroptosis to combat cancer treatment resistance. Conducting the same nano-strategies will significantly decrease the leukemia risk of recurrence. Various research directions should be investigated to reach this target. Nonetheless, the advances in the field of cancer nanomedicine still need to be approved clinically before becoming routine substitutes for conventional treatments. These nanomedicines are mainly concentrated on solid tumors. Although liquid tumors seem to be forgotten somehow, the recent nanomedicine approaches are of high potential to manage liquid tumors, both in circulation and in the local environments such as bone marrow, given that most malignant cells are spread throughout the body via blood and lymph. To get rid of those cancerous cells with specificity, we require more innovative strategies based on NPs. Ferroptosis is a novel regulated cellular death distinguished by highly iron-dependent lipid peroxidation. Many pieces of evidence reflect the association between ferroptosis and cancer. Selective ferroptosis induction can be viewed as a potential treatment approach for the treatment of cancers. Many studies looked for novel inducing reagents to launch ferroptosis in an efficient manner. Therefore, nanomedicine's introduction is the dawn of a new era for developing novel ferroptosis inducers for specific treatment of leukemia. Concurrently, advancements in the study and management of hematopoietic malignancy disorders may impact ferroptosis. However, the current study on ferroptosis in hematological malignancies is still in its early stages. Future studies will focus on confirming the impact and mechanism of ferroptosis on hematological malignancy cells through additional in vivo and in vitro studies.

## Data Availability

By ordering from the corresponding author.
